# The Primary Visual Cortex Is Differentially Modulated by Stimulus-Driven and Top-Down Attention

**DOI:** 10.1371/journal.pone.0145379

**Published:** 2016-01-05

**Authors:** Marek Bekisz, Wojciech Bogdan, Anaida Ghazaryan, Wioletta J. Waleszczyk, Ewa Kublik, Andrzej Wróbel

**Affiliations:** Department of Neurophysiology, Nencki Institute of Experimental Biology, Warsaw, Poland; University of Salamanca- Institute for Neuroscience of Castille and Leon and Medical School, SPAIN

## Abstract

Selective attention can be focused either volitionally, by top-down signals derived from task demands, or automatically, by bottom-up signals from salient stimuli. Because the brain mechanisms that underlie these two attention processes are poorly understood, we recorded local field potentials (LFPs) from primary visual cortical areas of cats as they performed stimulus-driven and anticipatory discrimination tasks. Consistent with our previous observations, in both tasks, we found enhanced beta activity, which we have postulated may serve as an attention carrier. We characterized the functional organization of task-related beta activity by (i) cortical responses (EPs) evoked by electrical stimulation of the optic chiasm and (ii) intracortical LFP correlations. During the anticipatory task, peripheral stimulation that was preceded by high-amplitude beta oscillations evoked large-amplitude EPs compared with EPs that followed low-amplitude beta. In contrast, during the stimulus-driven task, cortical EPs preceded by high-amplitude beta oscillations were, on average, smaller than those preceded by low-amplitude beta. Analysis of the correlations between the different recording sites revealed that beta activation maps were heterogeneous during the bottom-up task and homogeneous for the top-down task. We conclude that bottom-up attention activates cortical visual areas in a mosaic-like pattern, whereas top-down attentional modulation results in spatially homogeneous excitation.

## Introduction

Attention influences neuronal activity at the early stages of visual processing, including areas 17 and 18 as well as the thalamic nuclei (for reviews see [1,2]). Volitional attention is elicited by the task and acts via top-down signals from higher cortical areas, often without specific visual stimulation (i.e., during stimulus expectation—[3,4,5]). Other attentional mechanisms are evoked by bottom-up signals from salient stimuli [6,7]. The attentional effects found in these experiments include higher baseline activity and increased sensitivity to external stimulation (reviewed in [1,8]). Because the control of top-down and bottom-up attention involves different sensory and modulatory circuits [9,10,11], it may also differentially shape the gain mechanisms at the level of the primary visual cortices.

Attentional processes have been related to oscillatory activity of different frequencies (theta, alpha, beta, and gamma), and their specificity remains a matter of debate [1,11]. Our experiments on behaving cats showed that enhanced beta activity may serve as an attention carrier in cortico-thalamic loops involving cortical areas 17 and 18, the medial suprasylvian cortex, and the first- and higher-order thalamic nuclei [4,6,12]. We proposed that enhanced beta signals depolarize the input levels at all stages of visual processing via a frequency facilitation mechanism in feedback pathways and thus increase the gain within specific information channels [4,13]. Similarly, other researchers have found that oscillations at beta frequencies are involved in a top-down attentional searchlight from the frontal cortex [11,14,15,16,17,18], and in the processing of novel stimuli [19].

In this study, we used electrical stimulation of the optic chiasm to validate our hypothesis that attention-related beta activity changes cortical sensitivity [2,20]. We suspected that high- and low-amplitude beta oscillations would differentially change the functional excitability of primary cortical areas. Our results provide evidence in support of this hypothesis and show that the excitation map measured in the visual cortex depends on the type of attention. Whereas the exogenous (bottom-up) and endogenous (top-down) modes of attention were both accompanied by a significant increase of beta activity within primary visual areas, differences in the spatial organization of the cortical responses (evoked by chiasmatic stimulation during high and low amplitude beta signals) and in the internal cortical synchronizations within the beta frequency range were clearly evident between the two attentional situations. Thus, the functional organization of the visual cortex in the two modes of attention appeared to be shaped by different spatial maps of beta activity. These results suggest that different attentional requirements may set the specific functional organization of the visual cortex to optimize its function according to the actual information being processed.

## Materials and Methods

The experimental procedures were performed in accordance with the 86/609/EEC directive and were approved by the Local Ethical Commission at the Nencki Institute. All efforts were undertaken to minimize the animals’ suffering and the number of animals used.

### Behavioral paradigms

**Fig 1:**
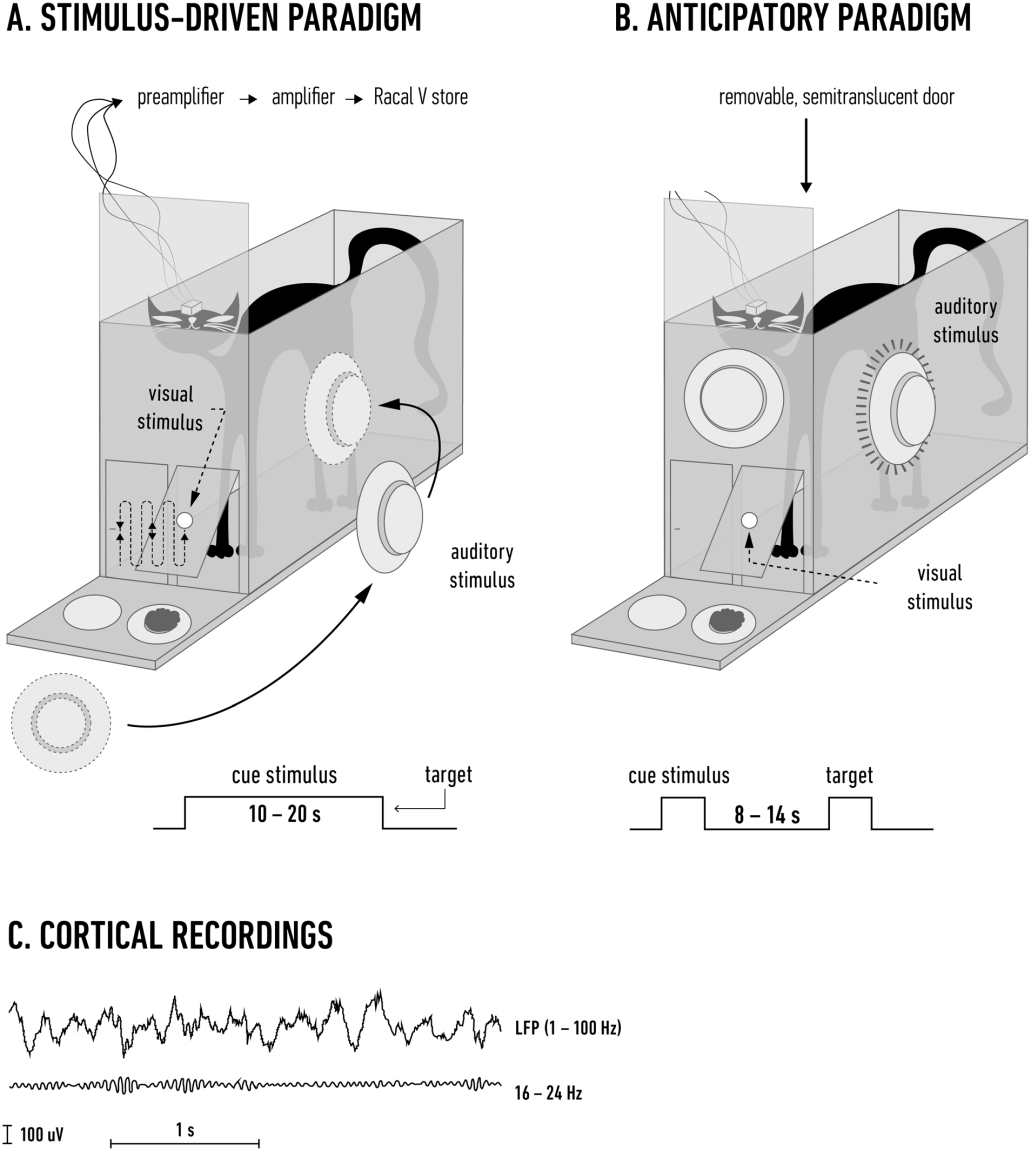
Experimental paradigms. A. In the stimulus-driven attention paradigm, the visual and auditory trials started with the cue stimulus (a light spot or a modulated noise delivered from a loudspeaker) that moved in front of the cage for 10–20 s. The spot was switched off in front of one of the doors or the noise stopped along the side wall. The transparent screen was lifted 2 s later, and the cat was allowed to open the indicated door for a food reward. Visual and auditory trials (n = 12–20 for each) were randomly intermingled. The cage was fully closed during the experiment by a removable cover (not shown). With the exception of the two semi-translucent doors on the front, the rest of the cage was opaque (the cat’s entire body was drawn only for graphical demonstration). B. During the anticipatory paradigm, the cue was either a short (1 s) diffuse flash on both doors or a white noise delivered from the front loudspeaker. The animal then anticipated the target for 8–14 s with no stimulation present, after which a small spot of light or white noise from the side loudspeaker was delivered. C. The upper trace shows a typical LFP recording in which the beta activity can be traced by eye; the filtered beta band of this record is shown beneath the LFP signal. See the Methods section for details.

Six adult, castrated male cats weighing 3–4.5 kg were used in the experiments. After habituation to the laboratory equipment and personnel, each cat was trained to discriminate the spatial position of a reward based on visual or auditory cues in intermingled trials. Training was performed in a wooden box (20 x 45 x 45 cm) with semi-translucent doors on the right and left of the frontal wall; feeders were placed behind these doors and used to administer a food reward. A transparent screen placed in front of the doors prevented immediate access to the feeders. This screen was moved up at the completion of a trial, allowing the animal to make a behavioral response and access the reward (Fig 1). The semi-translucent quality of the doors allowed the presentation of light stimuli but effectively filtered out any other visual clues, such as the presence of the food reward or movement of the loudspeaker in the auditory trials. The animals were trained under low mesopic conditions (0.05 cd/m^2^) for paradigms that required either "stimulus-evoked" (three cats) or “anticipatory" (four cats) attention; one animal was trained in both paradigms, starting with the anticipatory task. The training procedures were described in detail previously [4,6]. Briefly, visual stimuli were projected from outside the box onto the front of the semi-translucent doors (in both paradigms), and sounds were delivered through an external hand-held loudspeaker (in the stimulus-driven paradigm; Fig 1A) or through three loudspeakers attached to the front left and right wall of the training box (in the anticipatory paradigm; Fig 1B). In both paradigms, the visual and auditory trials were presented in a random order (24–40 trials in a daily session; 12–20 of each modality), and each trial started with a stimulus cue that signaled the modality of the trial.

In the stimulus-driven paradigm, the cue stimuli were presented for 10–20 s. In the visual trials, the cue consisted of a 0.5 x 1° light square (1.6 cd/m^2^ intensity) that moved horizontally back and forth across both doors (at a linear speed of approximately 5–10 cm/s), with simultaneous oscillations in the vertical plane (frequency 1 Hz, amplitude 4 cm). The cue for the auditory trials was a modulated noise stimulus (5 kHz center frequency; 50–55 dB; 1 Hz modulation rate). The sound was produced by an external loudspeaker that was turned on centrally approximately 25 cm from the front of the training box and subsequently moved behind one of the side walls. Both types of trials lasted 10–20 s. The animal was required to determine on which side the moving stimulus was switched off (left or right door for the visual stimulus and left or right wall for the auditory stimulus, (the target in Fig 1A) and open the door on the same side to receive a reward.

The anticipatory paradigm started with a 1 s cue—either a diffuse light flash (0.6 cd/m^2^) that was back-projected on the doors or white noise delivered from the loudspeaker in front of the box (60 dB). During the trials, the animal anticipated the target stimulus for 8–14 s (the delay was changed randomly) without any stimulus present. The subsequent visual target was a small light square (0.5 x 1°; 1.6 cd/m^2^ intensity; 1 s duration) that appeared on one of the doors, while the auditory target was a 1 s white noise (approximately 50 dB) delivered from a loudspeaker mounted behind the left or right wall; the target location indicated the door that should be opened for a food reward (Fig 1B).

The animal’s behavior was observed on-line (and video recorded for two cats). In both paradigms, the access to front doors was blocked throughout the trial by the transparent screen. Two seconds after the disappearance of the target stimulus, the screen was slid up and the appropriate door could be opened, allowing the cat to reach the food reward. The incorrect door remained locked. The cat’s attempt to open the wrong door was counted as an error, and in such cases, the correct door was immediately locked to prevent the cat from a second-choice response. Aside from the lack of a food reward, there was no additional penalty following an incorrect choice. Cats did not receive any food before the experiment in order to increase motivation. This was enough to run 24–40 trials, each reinforced by a small piece of meat, until the animals decreased their performance level. The time window for the animal’s response was restricted in both tasks to about 3 s and after three delayed attempts or behavioraly manifested disinterest the session was ended.

The learning procedure started with the visual task, and the auditory trials were not introduced until after the animal reached a 90% performance level on the visual task. The intensities of the visual and auditory target stimuli were identical in both paradigms. We determined that the animal had reached the behavioral criterion when it differentiated the location of the target stimulus in both the visual and auditory tasks with at least 90% accuracy over three successive training days. For individual cats, the period of time to reach criterion varied between 23 and 35 experimental days. Continuous observation of the animal's behavior allowed for proper evaluation of the cats’ responses and the exclusion of all trials during which the cats were grooming, moving or turning away from the front wall.

Our previous results indicated that the attentional beta mechanism was modality-specific. We observed enhanced beta power within the visual cortex during visual attention trials and within the auditory cortex during auditory trials [6]. Thus, the signals recorded in the visual cortex during auditory trials served as a control indicator for low beta activity.

### Animal preparation

The animals were prepared for electrode implantation after the completion of training. On the day preceding surgery, the cat was injected with dexamethasone (0.3 mg/kg, intramuscularly (i.m.); Dexaven, JELFA, Poland) to reduce brain edema. The implantation was performed after premedication with 0.2 mg/kg propionylpromazine, i.m. (Combelen, Bayern) and in most cases, under sodium pentobarbital anesthesia (20–35 mg/kg, intraperitoneally (i.p.); sodium pentobarbital solution; Vetbutal, Biowet, Puławy, Poland), with supplementary doses when required (3 mg/kg, i.m.). In two cats, similar premedication was applied, and then anesthesia was induced by a mixture of ketamine (20 mg/kg, i.m.; Ketanest, BioVet) and xylazine (3 mg/kg, i.m.; Xylavet, ScanVet) and maintained using isoflurane (1% in air; Aerrane, Baxter-Poland). Atropine sulfate (0.1 mg/kg, i.m., atropinum sulfuricum, Polfa, Warsaw, Poland) was given at the beginning of surgery to prevent excessive bronchial secretions. Two to four recording electrodes (tungsten wire coated with lacquer; 80–130 μm exposed tip; 50–100 kΩ impedance at 1 kHz) were implanted approximately 1 mm apart with the tips below layer 4 of the left visual cortex, in areas 17 and 18. The superficially accessible posterior locations representing the area centralis were chosen in each cortical area. The visual field representation of the recording site was estimated electrophysiologically during the implantation by recording the 'swish' to hand-held visual stimuli. The position of the area centralis was rechecked several times due to the lack of ocular muscle paralysis during the measurements. Electrode positions were subsequently confirmed during postmortem histological verification (see below). Two tungsten or stainless steel electrodes (0.2–0.4 mm in diameter; 10–20 kΩ impedance at 1 kHz) were stereotactically inserted into the optic chiasm for bipolar electrical stimulation; their tip positions were corrected by maximizing the amplitudes of electrically evoked cortical potentials (EPs). A stainless steel bone screw served as a ground and reference. All of the electrodes and a connecting plug were fixed to the skull using dental acrylic. After the surgery, the cats were left to recover under the supervision of the veterinary staff. Recordings started approximately two weeks post-surgery. At the conclusion of all recording sessions, small electrolytic lesions (5 μA for 15 s; positive DC) were performed to mark the electrode tip positions. The cats were then anesthetized with an overdose of sodium pentobarbital (Vetbutal) and perfused with saline followed by 4% paraformaldehyde in 0.1% phosphate buffer. The brains were removed and then stored in the same fixative for subsequent histological verification. The physiologically estimated retinotopic positions of the recording sites were correlated with postmortem anatomical and histological examination.

Each electrode was classified as located in area 17 or area 18 by (i) comparing the location of the electrode marks on cortical surface with the visuotopic maps of Tusa et al. [21,22] and (ii) on the basis of the electrode traces in the Nissl stained coronal slices where thicker layer 3 with large pyramidal somata and thinning of layer 4 indicated transition to area 18 [23,24]. These positions are listed in Table 1.

**Table 1:** Electrode location and corresponding retinotopic position in the experimental animals.

Cat	Paradigm	Area 17/electrode	Area 17 RF (Az/El)	Area 18/electrode	Area 18 RF (Az/El)
A	S-D	Cx17/1	5/-5	Cx18/1	20/-10
				Cx18/2	5/-4
B	S-D	Cx17/1	0.5/+2.5		
		Cx17/2	2/0		
		Cx17/3	-1.5/-2		
F	S-D / Ant			Cx18/1	5/-5
				Cx18/2	5/-10
C	Ant	Cx17/1	0/+1		
		Cx17/2	0/-2		
		Cx17/3	-0.5/-10		
D	Ant	Cx17/1	0/-2.5	Cx18/1	5/-5
		Cx17/2	0/-5	Cx18/2	5/-10
E	Ant	Cx17/1	0/-5	Cx18/1	5/-15
		Cx17/2	5/-10	Cx18/2	20/-15

Az—azimuth (values given in degrees). El—elevation. S-D—stimulus-driven attention paradigm. Ant—anticipatory attention paradigm. RF—receptive field.

### Recording and data analysis

**Fig 2:**
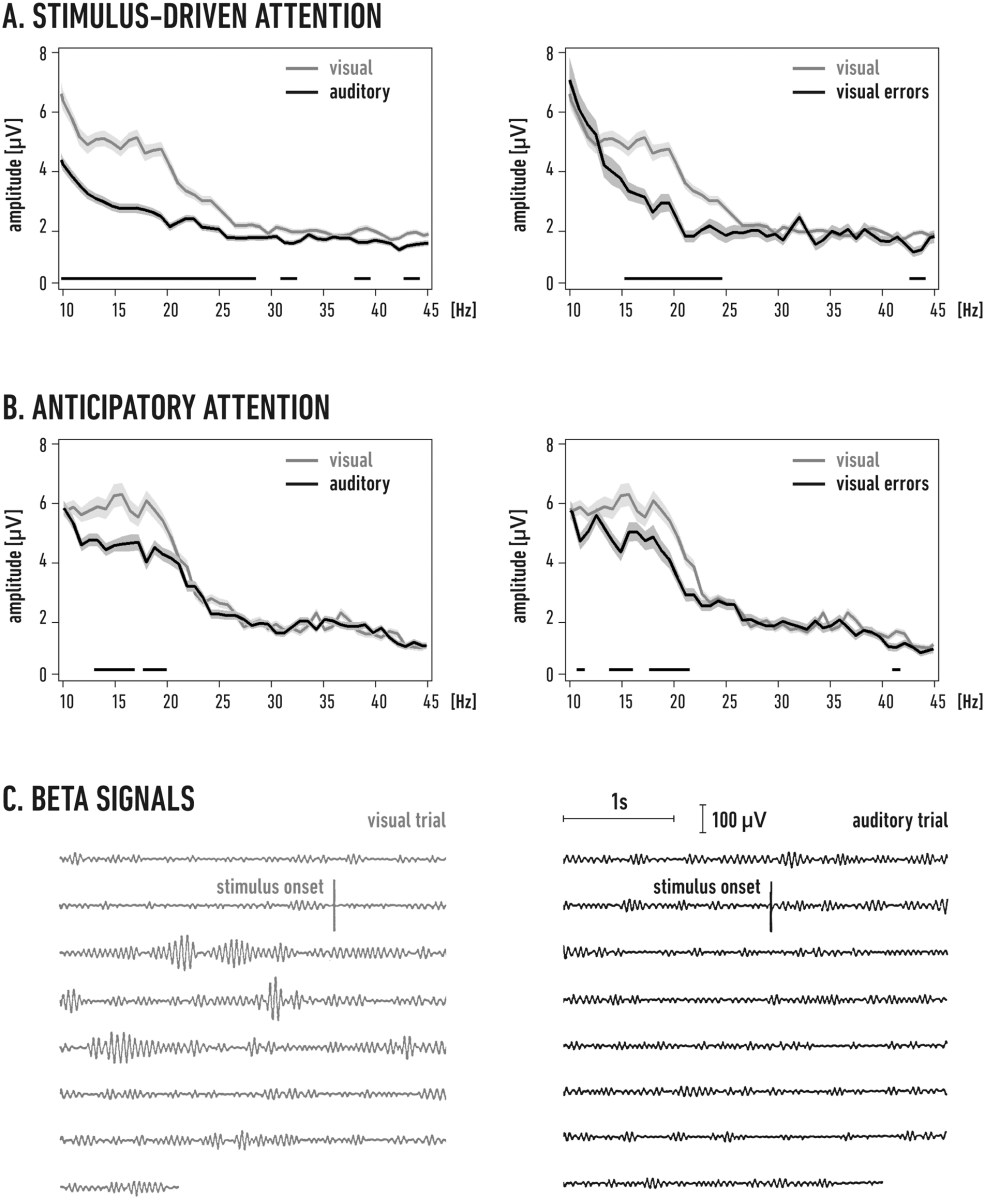
Beta activity increases in the primary visual cortex during attention trials. A. Stimulus-driven paradigm (cat B, Cx 17/1, see Table 1). Left: mean FFT spectra obtained from all correct trials during the three experimental days after the behavioral criterion was reached. The visual (gray) and auditory (black) means were obtained by averaging respectively 820 and 760 spectra of 1.28 s long data epochs (see Methods for details). Right: comparison of the mean spectrum from the correct visual trials (gray; same as in the left panel) with the mean FFT from 90 epochs from incorrect trials (black). B. Anticipatory paradigm (cat E, Cx 17/2). The recorded signals were subjected to the same analysis as in A. Means were obtained from 748 and 765 spectra for correspondingly correct visual and correct auditory trials, and from 320 spectra for incorrect visual trials. In A and B, the shading around the FFT spectra indicates the SEM. The lines above the horizontal axes denote the frequencies at which significant differences were found between the spectra from the correct visual and auditory (left) or correct and incorrect visual (right) trials (Student's t-tests with FDR correction for multiple comparisons, see the Methods section; P ≤ 0.05). An example of filtered (16–24 Hz) beta signals recorded from cat B at the beginning of two correct stimulus-driven visual and auditory trials (right and left panel, respectively) performed on the second day after the cat reached the behavioral criterion. The consecutive horizontal lines in each panel represent a continuous signal. The vertical lines separate the final part of the signal corresponding to the inter-trial period preceding stimulus onset and the start of the stimulus presentation during the trial.

Monopolar cortical local field potentials (LFPs) amplified ×1000 (bandwidth 1 Hz to 5 kHz) were recorded throughout the entire experimental session on an FM magnetic tape recorder (Racal V-Store; Racal Recorders, Southampton, UK). For FFT and correlation analysis LFP data were low-pass filtered (3 dB amplitude attenuation at 100 Hz; 24 dB/octave; Bessel analog filter) before digitization at a 400 Hz sampling rate. To analyze the evoked responses to optic chiasm stimulation, full-bandwidth LFP signals were digitized at 20 kHz and stored using an AD converter (Power 1401) and data acquisition software (Spike2) from CED (Cambridge, UK). Additional markers for the stimulation pulses were acquired as time events. Custom scripts in Spike2 and MATLAB (Mathworks, USA) were then used to analyze the recorded signals. The signal analysis was limited to LFPs recorded during the cue (10–20 s in the stimulus-driven trials) or between the end of the cue and the beginning of the target (8–14 s during the anticipatory trials). Note that for the anticipatory paradigm, this period was devoid of any added sensory stimulation. Frequency spectra were calculated over a number of consecutive time epochs for each trial using fast Fourier transform (FFT; e.g., Fig 2). We used 1.28 s epochs (512 samples) with a 240 sample overlap. The raw data within the epochs were multiplied by the Hanning window function before the Fourier transformation. Mean spectra were generated by averaging FFTs over all of the artifact-free data epochs from the trials of a given modality that ended with the same (correct or incorrect) behavioral response. Means were calculated separately for each task, animal, and recording site, based on the signals recorded during three consecutive days (after the cat reached behavioral criterion). For each mean single-site spectra, the FFT amplitude values were further averaged over the beta range (16–24 Hz); this average was termed the beta FFT amplitude.

To compare the results obtained for different electrodes and animals, we calculated the normalized percentage difference between the beta FFT amplitudes for the visual and auditory trials. Thus, for each recording site, the beta FFT amplitude obtained for the auditory trials was subtracted from that obtained for the visual trials; the difference was divided by the respective beta amplitude of the auditory FFT and multiplied by 100%. The result of this calculation is referred to below as the “relative visual beta amplitude” and describes percentage difference between the visual and auditory beta amplitude.

For the analysis of correlations, the LFP signals from trials without electrical stimulation were digitally band-pass filtered with half-amplitude cutoff frequencies of 16–24 Hz. The cross-correlation function was calculated between the filtered signals recorded from any two cortical sites in each animal. The technical details for calculating the normalized cross-correlations (values between -1 to +1) for single trials can be found elsewhere [25,26]. The cross-correlations were calculated with time shifts from -1 to +1 s and bins of 2.5 ms. For each pair of electrodes, single-trial cross-correlations were averaged over all trials (correct response, artifact free) of the same modality (visual or auditory) that were executed during the three days following the attainment of behavioral criterion. We defined the “visual and auditory correlations” as the maximal values of the averaged cross-correlations.

**Fig 3:**
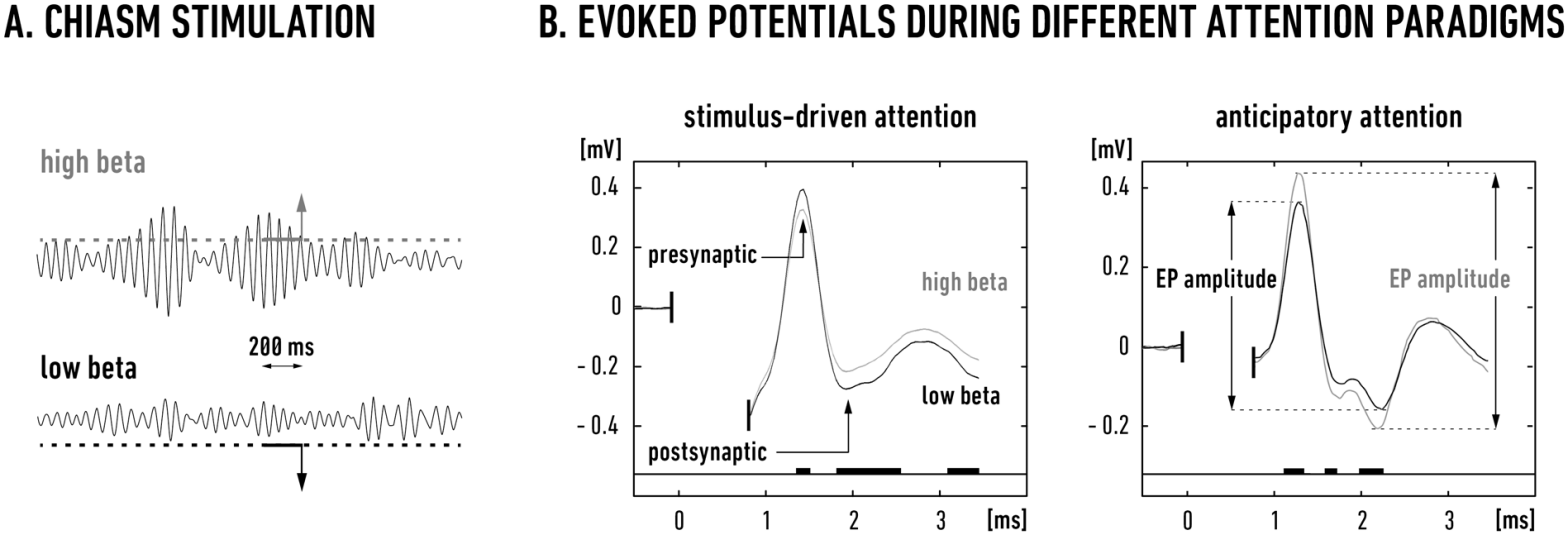
Summary of evoked potentials analysis. A. The EPs recorded during visual trials were clustered off-line to either high beta or low beta groups based on the strength of the digitally band-pass filtered beta signals (16–24 Hz) preceding chiasmatic stimulation (see the Methods section). The drawing presents two exemplary sweeps of these filtered signals with the corresponding high and low beta amplitudes. The threshold values (dotted horizontal lines) were set based on the average amplitude of the beta signal from all visual trials on a given experimental day. The vertical arrows (matched in time for illustration purposes) indicate the timing of two chiasmatic stimulations that were preceded by 200 ms of the eruption (upper sweep, gray horizontal line) or depression (lower sweep, black line) of beta oscillations. B. Examples of the averaged EPs (recorded for the two behavioral paradigms in two different animals) calculated from the clustered data after beta depression (black lines; means were obtained from 43 clustered EPs in the stimulus-induced and from 32 in the anticipatory task) or beta eruption (gray lines; means were obtained from 47 clustered EPs in the stimulus-induced and 35 in the anticipatory task). Electrical stimulation of the optic chiasm was delivered at time zero (artifact removed from the picture). The thick black horizontal lines denote significant differences between the corresponding values of the two mean EPs at the same latency (Student's t-tests with FDR correction for multiple comparisons; P ≤ 0.05). Note that during the high beta state, the amplitudes of the cortical EPs decreased in the stimulus-driven attention paradigm (cat B, VCx 17/2), and increased in the anticipatory attention paradigm (cat C, VCx 17/3).

EPs were recorded in response to electrical stimulation of the optic chiasm (50–150 μA; 0.2 ms cathodal pulses), which was repeated at random intervals from 1 to 3 s (mean 2 s; Fig 3A) throughout the entire duration of a trial. The stimulation current was set to approximately 2-3x threshold (approximately 25–75 μA) to evoke a clear response but without producing any noticeable behavioral reactions. The first cortical response after chiasmatic stimulation was a large population spike, which could be attributed to the synchronous volley of the lateral geniculate nucleus (LGN) afferent fibers (presynaptic bipolar wave, < 2 ms post stimulus, Fig 3B). The falling phase of this wave was further deepened (at approximately 2.5 ms), likely due to the current sink created by the postsynaptic responses of cortical neurons receiving input from Y class LGN relay cells (cf. [27,28]). The amplitude of this postsynaptic field is related to the number of synchronized excitatory and inhibitory postsynaptic potentials (EPSPs and IPSPs, respectively), their average sizes, and the actual polarization of the neuronal membranes (cf. [29,30]). The second wave (> 3 ms; partially truncated in Fig 3B) is likely due to PSPs evoked in cortical cells innervated by X relay LGN cells, as their fibers are slower and have a higher stimulation threshold in the nerve and a wider range of latencies in cortical recordings. In many of our recordings this presumably X-related field component was negligible due to low stimulus intensity, which was just above the threshold for the Y pathway (for details see [29]). Therefore, in our analysis the EP amplitude was measured as a voltage difference between the highest positive post-stimulus wave at approximately 1.5 ms (incoming spike volley) and the lowest post-stimulus potential at approximately 2.5 ms, representing the extracellular field of postsynaptic potentials (PSPs) from mostly Y channel inputs.

For the EP analysis, we extracted 1.5 s data epochs starting 1 s before the stimulation pulse. The baseline voltage level was calculated from 25 samples preceding the stimulation and was then subtracted from the data in each epoch. To quantify the relationship between ongoing cortical beta activity and EP amplitude, we clustered the EPs based on the strength of the digitally band-pass filtered (16–24 Hz) beta signals before the stimulus. The chosen band encompassed the beta frequencies that were enhanced during the visual task (see lines of significance in Fig 2A and 2B). For each electrode, the beta signals during the 200 ms period (between 300 ms and 100 ms) preceding the moment of stimulation were assumed to be ‘enhanced’ (“high beta”) or ‘reduced’ (“low beta”) if their amplitudes were 25% higher or 25% lower, respectively, than the average beta signal amplitude calculated from all of the visual trials on a given experimental day (Fig 3A). We assumed that high beta signals preceding the EP would influence its amplitude differently than low beta signals [31,32] due to cortico-cortical recurrent pathway with build in frequency potentiation mechanisms [2,4]. The beta activity during the last 100 ms before stimulus delivery could not be included into the averaging period due to the filtering window algorithm which distorted this part of the signal by inclusion of the stimulation artifact. We hypothesized, however, that the potentiation mechanism in the cortical loops should last along this period (adopted from duration of frequency facilitation measured for cortico-geniculate branch [13]), and this assumption was confirmed by the obtained results. Single EPs preceded by “high beta” or “low beta” signals were grouped according to the experimental paradigm (stimulus-driven or anticipatory). For individual recording sites in each animal, the data from at least three recording days were then separately averaged (Fig 3B). To calculate the group average for all animals, we first calculated the normalized percentage differences of the EP amplitudes for the individual electrodes by subtracting the amplitude values obtained in a “low beta” state from those measured during a “high beta” state, dividing the result by the “low beta” amplitudes and multiplying it by 100%. As in the case of the FFT analysis, normalization was used here to compare the effects obtained for the different electrodes and animals. The resulting relative value describes the percent difference of the “high beta” EP amplitude from the “low beta” EP amplitude. Finally, the normalized differences in the EP amplitudes were averaged for the entire set of electrodes from all the cats trained in the same paradigm.

The exemplary raw LFP recordings are available at direct link: https://cloud.nencki.gov.pl/f/866486cf63/?raw=1.

### Statistical analysis

Significant differences between corresponding values at the same frequency in two averaged FFTs or between corresponding values at the same latency in two averaged EPs were estimated with an unpaired Student’s t-test (as the visual and auditory trials could not be directly matched). In both cases, corrections for multiple comparisons were calculated using the False Discovery Rate (FDR) method according to Benjamini and Hochberg [33]. All averaged data are presented as the mean ± standard error of the mean (SEM). The significance of differences between the mean values was assessed with an unpaired or paired Student's t-test. The significance of differences between the mean values and zero was checked with one-sample t-test. Differences between variances were tested using the F-test. In the case of the beta signal correlations, the Holm-Bonferroni correction was used for multiple comparisons. The differences were regarded as significant when P ≤ 0.05 (two-tailed criterion). The statistical analyses were performed in Prism 6 (GraphPad Software Inc., USA) and MATLAB (Mathworks, USA). In the text we gave the exact values of P until they were very small (less than 0.001). In such situation the notation P<0.001 was used. In a few cases in which we reported more that one comparison, we indicated the maximal P threshold that was adequate for all.

## Results

### Electrode locations

Recording electrodes were placed in the left visual cortex, mostly close to the representation of area centralis, which means that they were often within transition zone between area 17 and 18. However, we classified such central electrodes as belonging rather to area 17 or 18 (see Methods for verification approach and Table 1 for electrode positions). Precise estimation of the recording depth was impossible due to build up of glial scars after about half a year of training and recordings. The position of the majority of the electrode tips was estimated to be below layer 4 and the full range was from layer 4 to 6. What is important is that groups of animals performing stimulus-driven and anticipatory paradigm did not differ in laminar distribution of recording sites and median level, which was at the border between layers 5 and 6.

### Attentional beta activity in the visual cortex

The primary visual cortices exhibit increased beta activity during bottom-up and top-down visual attention tasks compared with auditory tasks [4,6,12,34]. This was confirmed in the present sample of signals recorded from all sites investigated in primary areas 17 (12 locations; e.g., Fig 2A and 2B) and 18 (9 locations; not shown). The enhanced activity in the beta band resulted from an increase in the amplitude and frequency of beta bursts in the visual trials that ended with correct responses and was observed between the cue and target stimuli (Fig 2C). Note that in the case of the anticipatory paradigm, this time period was devoid of any added stimuli, demonstrating that the increased beta activity was not related to visual stimulation.

Our main argument in support of the hypothesis that beta activity serves as an attention carrier [2,20] relies on the observation that only successful (and not incorrect) visual trials were accompanied by significantly enhanced beta activity (Fig 2A and 2B, left panels) in both the stimulus-driven and anticipatory paradigms. The LFP signals recorded during the incorrect visual trials contained significantly less beta activity and were similar to correct auditory trials (comp. left and right panels in Fig 2A and 2B). However, we observed some residual beta activity that appeared in the incorrect visual trials and correct auditory trials when the animal was waiting for a target without external visual stimulation (Fig 2B). Such beta signals occasionally increased during the several-seconds-long trials that lacked external stimulation, which may have been due to the accidental wavering of visual attention. Indeed, we have found that during 10–15% of the incorrect visual anticipatory trials (or correct auditory anticipatory trials), the signals recorded from the visual cortex contained a substantial amount of beta activity (S1B and S1C Fig). Conversely, a similarly small proportion of the correct visual trials in this experimental paradigm were not accompanied by high beta activity (see S1A Fig).

Nevertheless, the beta FFT amplitudes (see the Methods section for details) measured during the visual trials for all recording sites in areas 17 and 18 were significantly larger than those obtained for the auditory task for both the stimulus-driven (on average by 20.3 ± 3.9%, n = 8 sites; P < 0.001) and anticipatory (on average by 6 ± 1%, n = 13 sites; P < 0.001, single group t-tests, S2 Fig) paradigms, confirming the visual attention-related increase in beta activity in the two tasks. Notably, the stimulus-driven visual attention, on average, enhanced the beta activity more strongly (relative to the auditory task) than the anticipatory visual attention (P < 0.001, t-test). This could not result from higher motivation or difficulty of the task as behavioral measures within both groups were not different (average times to criterion were 32 ± 2 and 31 ± 3 days, average ratios of incorrect responses were 7.4 ± 1.7%, and 7.2 +/- 2.1%, respectively for stimulus-driven (n = 3) and anticipatory (n = 4) groups; P > 0.9 for both comparisons, t-tests).

Moreover, analysis of the LFPs led us also to conclude that the relative increase of beta activity during the visual trials in the stimulus-driven task was characterized by 18 times higher variance between the values obtained for the different electrodes, compared to the anticipatory situation (the corresponding variances were 0.018 vs. 0.001; P < 0.001, F-test; compare the box plots in S2 Fig). This indicates that there were much larger differences in the magnitudes of the visual beta signals between different recording sites (spatial heterogeneity) in the stimulus-driven task. Conversely, the beta activity that was enhanced by endogenous attentional demands during the anticipatory visual trials appeared to be more even (i.e., homogeneous) throughout the visual cortices than the beta activity related to exogenous, stimulus-driven attention.

### The relationship between EP amplitude and ongoing cortical beta activity

It had been shown before that ongoing cortical activity modulates potentials evoked by peripheral stimulation in the cortex [31,32] and we expected that high- and low-amplitude beta oscillations could differently affect cortical EPs. The average amplitudes of EPs recorded during the visual and auditory trials in all animals appeared to be similar (P = 0.2, t-test). This was not surprising because random stimulation may only rarely coincide with the relatively short (100–350 ms) oscillatory beta bursts ([26]; cf. Figs 1C and 2C). To determine whether cortical beta bursting changes the excitability of cortical neurons, it was necessary to select EPs that were preceded by high- or low-amplitude beta signals.

To achieve this, we selected and averaged the EPs according to the mean amplitude of the beta signal during a constant 200 ms time window preceding chiasmatic stimulation (for details, see the Methods section and Fig 3A). This procedure allowed us to compare the amplitudes of average EPs evoked after high-amplitude beta bursts (termed the “high beta” state) with those evoked after low-amplitude beta bursts (“low beta” state).

**Fig 4:**
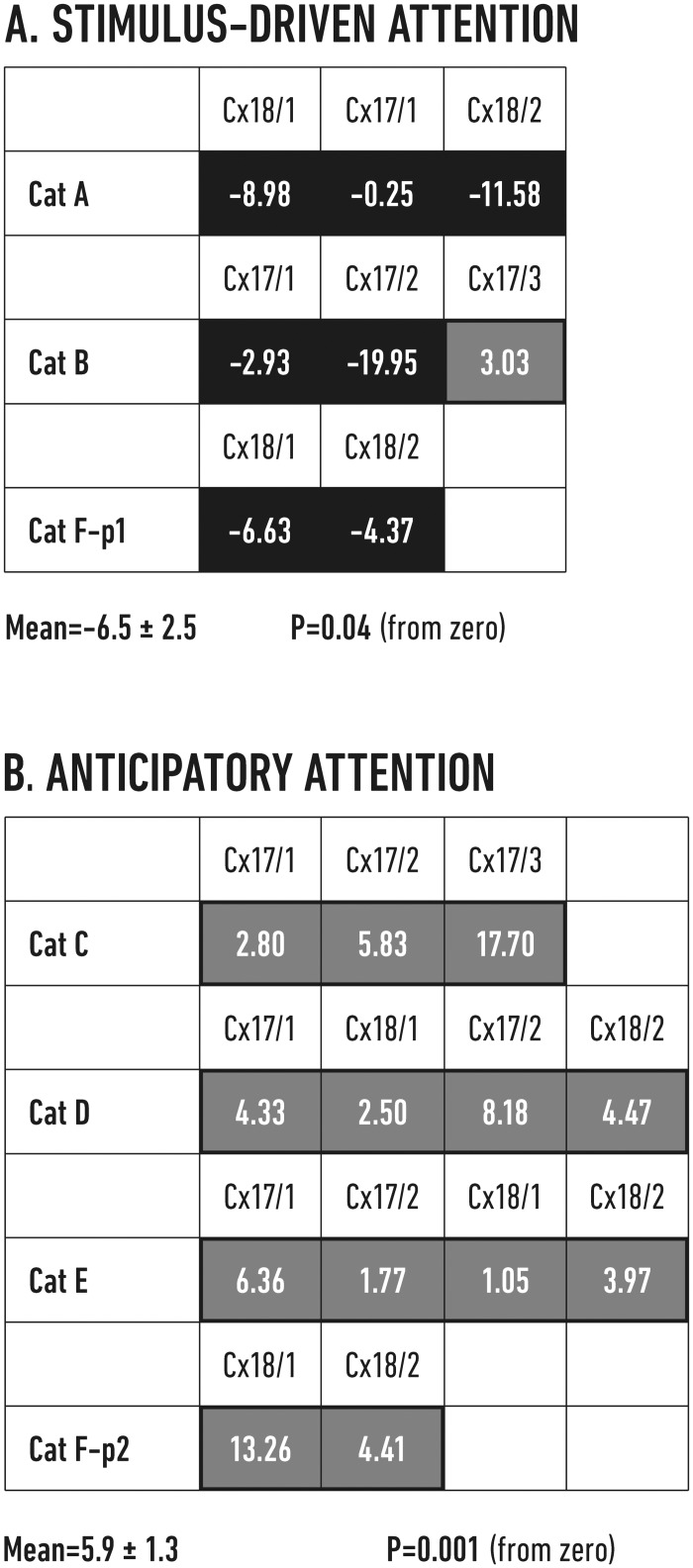
Relative amplitude differences of the evoked potentials during high and low beta states in visual trials. EPs induced by chiasmatic stimulation during visual trials were analyzed in all recording sites and all cats in the two experimental paradigms (cf. Table 1)—A stimulus driven attention, B—anticipatory attention. Each box shows the normalized percentage difference (Δ%) between the EP amplitudes calculated for the appropriate recording site: Δ% = 100% x (high − low) / low, where “high” indicates the mean amplitude of the EPs that were preceded by bursts of high beta activity and “low” refers to the mean amplitude of the EPs that were preceded by low beta activity. Black boxes = high < low, gray boxes = high > low. Below each table we present mean amplitude change (with SEM) obtained in the relevant paradigm from all available sites and significance of its difference from zero (as P value from one-sample t-test).

Typical examples of such EPs obtained during visual trials of the stimulus-driven attention paradigm are depicted in the left panel of Fig 3B. It is notable that on average EPs that were preceded by bursts of high beta activity had lower amplitude than those preceded by low beta activity. This was true for all but one recording sites as depicted by the map of the normalized amplitude differences in Fig 4A. The average EP amplitude measured in visual trials of the stimulus-driven paradigm during high beta states was 6.5 ± 2.5% lower than the value obtained during the low beta periods (P = 0.04, one-sample t-test for a difference from zero).

Different results were obtained during the visual trials of anticipatory paradigm as visualized in the right panel of Fig 3B. In this situation the recorded EPs exhibited larger amplitudes in the high beta state (gray trace) than during the low beta state (black trace). This observation appeared to hold true for all the recording sites in all four cats, as shown by the map of the normalized amplitude differences in Fig 4B. Over all recording sites, the EP amplitudes obtained for the high beta state in the anticipatory paradigm were homogeneously higher (by an average of 5.9 ± 1.3%) than the values obtained for the low beta periods (P < 0.001, one-sample t-test for a difference from zero). It is notable, that the mean value of normalized differences of the EP amplitudes recorded by all electrodes during stimulus-driven task was negative and mean value obtained for the anticipatory task—positive (Fig 4A and 4B) and this difference was significant (P < 0.001, two-sample t-test).

These results showed that high vs low cortical beta activities changed the EP amplitudes in opposite direction depending on the attentional paradigm used. Taking into account that beta activity was in general smaller in auditory than in visual trials (Fig 2) we further compared EP amplitudes recorded during high-beta states of visual trials with those measured during low-beta states of auditory trials. This comparison showed even greater difference between mean EPs recorded in visual cortex (stimulus driven -9.6 ± 2.9, anticipatory 9.8 ± 2.3; P = 0.01 and 0.001 respectively, one-sample t-tests; see S3 Fig) than that described above for high and low beta in the same visual modality. Thus, our results confirmed that beta-related amplitudes and distribution of EPs recorded in visual cortex with peripheral stimulation substantially differ during stimulus-driven and anticipatory paradigms.

### Correlation of beta signals across different recording sites

**Fig 5:**
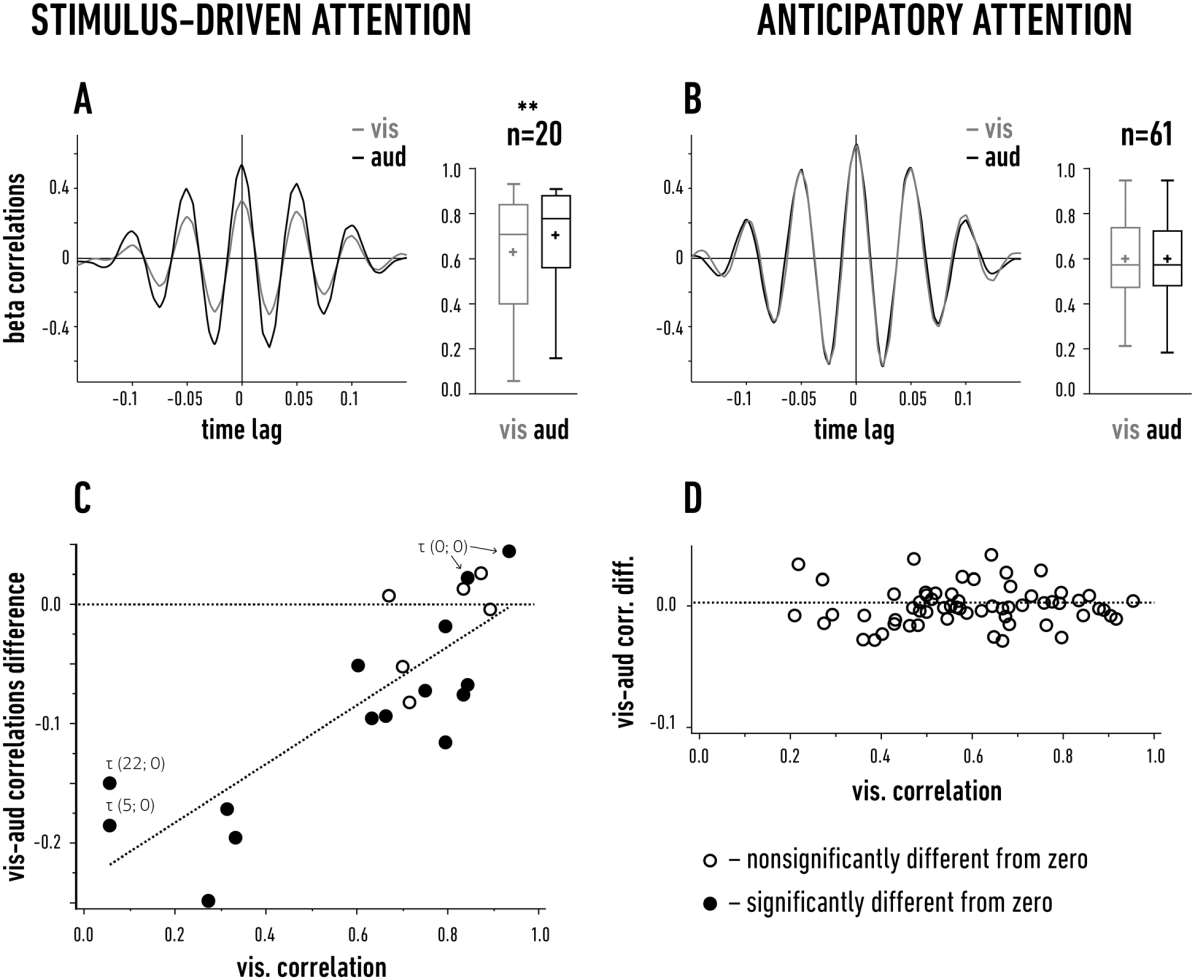
Synchronization of beta activity within the visual cortex. A, B. The representative cross-correlograms (left) and box plots of the maximal cross-correlation values (referred to as correlation) between the beta signals recorded at different sites in the visual cortex during the stimulus-driven (A) and anticipatory (B) attentional paradigms. The data from all pairs of recordings from area 17 and 18 were grouped together (n—number of site pairs). Gray box plots depict data obtained for the visual task (vis); and black box plots, for the auditory task (aud). The bottom and the top of the box represent the first and the third quartiles of the data range, respectively. The whiskers indicate the minimum and maximum of the data. The median and the mean values are shown by the horizontal line and the plus sign inside the box, respectively. Significant differences between the visual and auditory mean values during the stimulus-driven paradigm are denoted by ** (P = 0.002, t-test). C, D. The differences between the visual and auditory correlations obtained for individual pairs of sites are plotted against the respective visual correlation values in the stimulus-driven (C) and anticipatory (D) situations. Filled symbols indicate significant correlation differences for the individual electrode pairs (P ≤ 0.05, t-test). In the case of four pairs of electrodes, the time lag values (τ) in ms are shown in brackets for the visual and auditory cross-correlograms, respectively (see the text for details).

To further explore any possible differences in the cortical beta activation maps between the two attentional paradigms, we compared the spatial distributions of the beta correlations between different cortical sites. For this purpose, we calculated the normalized cross-correlations [25,26] between the filtered beta signals recorded from all of the cortical electrodes in each cat (examples shown in the left panels of Fig 5A and 5B; see the Methods section for details). We used maximal cross-correlation values as a measure of correlation strength (referred to in the following text as correlation). The resulting values for all electrode pairs are presented as box plots in Fig 5A and 5B (see also the detailed results showing the comparison of the correlations within and between areas 17 and 18 in S4A and S4B Fig).

During the stimulus-driven attentional paradigm, the mean correlation values in the beta range (16–24 Hz) obtained for all electrode pairs (in areas 17 and 18) during the correct visual trials (0.63 ± 0.06; Fig 5A, gray boxes) were smaller than those calculated during the (correctly performed) auditory task (0.71 ± 0.05; Fig 5A, black boxes), and this difference was highly significant (n = 20 electrode pairs, P = 0.002, paired t-test; see also S5 Fig). Thus, although the power of the beta signal in the visual cortex was larger for the visual trials compared to the auditory trials during the stimulus-driven attentional paradigm (cf. Fig 2A, left), the beta activity was more synchronized over the entire visual cortex during the auditory trials, when it remained in a non-attentive state. It should be noted that most of the increase in beta activity was not related to the processing of the visual stimulus but reflected attentional activity, as we have argued above. Accordingly, the observed differences in the beta correlation values should reflect the extent and organization of attentional modulation and not the specific processing of visual stimulus features. To confirm this hypothesis, we additionally calculated the mean correlation during incorrect visual trials (0.7 ± 0.04) for all pairs of recordings in the stimulus-driven task. Despite the presence of a visual stimulus, this correlation appeared significantly larger than the correlations given above for the correct visual trials (P = 0.009, t-test) and did not differ from the correlation calculated during the correct auditory trials (P = 0.5, t-test). This result indicates that the cortical beta correlations depended primarily on the level of attention and not (or only marginally) on visual stimulation.

A weak correlation measured for single electrode pairs during the visual task was accompanied by a substantially higher correlation during the auditory task, and this difference diminished as the visual correlations increased (Fig 5C). Thus, the overall differences between the visual and auditory correlations (calculated for single electrode pairs) plotted against the visual correlation showed a positive linear relationship (Fig 5C; Pearson r = 0.94, P < 0.001, mainly based on the data from area 17, as shown in S4 Fig). This relationship did not hold for the data from the incorrect visual trials, despite the presence of the stimulus (r = 0.21, P = 0.38; S6 Fig). This again confirms that the correlation pattern of the activated domains in the visual cortical areas that appeared during the correct trials during the bottom-up task indeed resulted from attentional modulation and not from the presence of the visual stimulus.

We noted that the linear relationship in Fig 5C included two-electrode pairs with highly correlated beta signals (> 0.8), for which the calculated correlations were significantly higher during the visual trials than the auditory trials. This supports the notion that such signals were recorded from functionally linked cortical domains, which exhibited increased coupling due to visual stimulation. The analysis of the correlogram time lags (the temporal position of the cross-correlation maximum with respect to the cross-correlogram center, [25]) that was calculated for pairs of signals at the two extremes of the relationship presented in Fig 5C (< 0.1 and > 0.8 values of visual beta correlations, marked by τ) also supports this view. Zero time lags were obtained for sites with highly correlated (> 0.8) signals (in both the visual and auditory trials), whereas non-zero time lags (i.e., 5 and 22 ms lags in Fig 5C) were obtained for relatively weakly correlated (< 0.1) recording pairs in the visual trials, with significantly larger beta correlation values during the auditory trials than the visual trials. We posit that the signals from electrode pairs with significantly higher beta correlations in the visual trials compared with the auditory trials in Fig 5C (filled dots representing significant positive differences in beta correlations) that had zero time lags during the visual trials belong to the same assembly of functionally linked regions in an otherwise heterogeneous beta activity map. In contrast, the small beta correlation values and non-zero time lags obtained for the visual attention trials support the notion that such signals were recorded from electrodes placed (by chance) in different beta domains of a heterogeneous functional mosaic.

The correlations calculated for the anticipatory paradigm differed in many respects from those obtained for the stimulus-driven paradigm. In the previous section, we concluded that the enhanced beta activity was more evenly distributed throughout the visual cortices during endogenously evoked visual attention than during a stimulus-driven task. In accordance with this observation, all of the beta band correlation coefficients during anticipatory attention ranged between 0.2–0.95 (Fig 5B) and were similar during both the correctly performed visual and auditory trials (0.59 ± 0.02 and 0.6 ± 0.02, respectively; n = 61 electrode pairs; P = 0.21, paired t-test), suggesting a uniform spatial correlation pattern of the beta signals (see also S5 Fig). Correspondingly, the differences between the visual and auditory correlations calculated for single electrode pairs varied around zero and were not related to the visual correlation values (Fig 5D; Pearson r = 0.003, P = 0.98). Importantly, none of the single-pair correlations in Fig 5D showed a significant difference between the visual and auditory trials, whereas many of the small-magnitude correlation differences appeared to be significant during stimulus-induced attention (compare Fig 5C). This suggests that there is no spatial difference between the functional organization of the visual cortices during the visual and auditory trials for the anticipatory attention paradigm, although the amplitude of beta activity was significantly higher during the visual task (Fig 2B).

## Discussion

In this experiment, we confirmed our previous findings that the beta frequency activity recorded in primary visual areas (17 and 18) is higher during bottom-up (stimulus-driven or exogenous) and top-down (anticipatory or endogenous) visual attention tasks than during corresponding auditory tasks (reviewed in [2]). Furthermore, we showed that the enhancement of beta activity during the visual attention tasks was paradigm-specific, i.e., the beta activity distribution over the visual cortex was heterogeneous in the stimulus-driven attentional mode and homogeneous during stimulus anticipation. This difference in the spatial organization of beta activation was confirmed by three independent analyses. We showed that the variability of the beta signal amplitudes between the individual recording sites was higher during exogenous attention (S2 Fig) and that the influence of beta signals on peripherally evoked cortical EPs (Fig 4) as well as the correlation of beta signals between pairs of cortical recordings (Fig 5) were heterogeneous in exogenous and homogeneous in endogenous attention. The difference in the spatial distributions of beta activities in both tasks could not be due to different motivation levels or task difficulties as behavioral measures (time to criterion and ratio of erroneous responses) were the same. Neither could it result from the systematic differences in the electrode locations in the cats performing different tasks since the distribution of the recording point in areas 17 and 18 and in cortical depth were similar in both experimental groups. We assume that the LFP signals recorded in our experiments largely reflect the activity of pyramidal neurons, given that other neuronal classes produce smaller electrical dipoles. The placement of the large electrodes in the lower cortical layers sums the activity mainly from infra- but also from supragranular pyramidal cells [35]. This positioning of the electrodes ensured effective recordings but disallowed the credible estimation of sources of beta signals. However, in accordance with our findings some research has indicated that high-amplitude beta activity is primarily recorded from infragranular layers [36,37,38,39,40].

The present results confirm our idea [6] that the beta frequency facilitation mechanism described originally for the cortico-geniculate projection in anaesthetized animals [13] serves as an attentional carrier in visual tasks. We have also previously proposed [2,4,20] that a similar role might be exerted by other feedback pathways that cascade back from higher visual centers (comp. reviews [1,41]). The results of other experimental studies are in accordance with this hypothesis [15,16,17]. In addition, our new data show that the exogenous and endogenous attention paradigms activate recurrent routes of the visual system in different manners.

### Stimulus-driven attention

It is generally agreed that sensory stimulation depolarizes restricted domains in the primary cortex involved in processing a salient stimulus. The processing involves the shaping of these domains by surround suppression, particularly by lateral inhibitory mechanisms and their sensitization via feedback projections from higher centers [42,43,44,45,46,47,48,49,50,51]; see [52] for review. A number of experiments have shown that the lateral extent of the sensory response is reduced during arousal and active behavior that requires stimulus-driven attention [53,54,55,56,57,58].

The shift to an attentional operating mode driven by the salience of the visual stimulus is well demonstrated by our chiasmatic stimulation, which probed the excitability of the visual cortex to incoming information. We posit that bottom-up visual signals activate lateral inhibitory circuits in the LGN and visual cortex that consequently result in smaller cortical EPs (Fig 3B, left; Fig 4A). These inhibitory circuits may shape the ongoing spatial pattern of cortical activation and produce relatively restricted high-correlation domains on a widespread background of weaker correlations [39,59,60,61].

This mosaic of functional organization of cortical activity would explain why the two-electrode pairs that were likely fortuitously implanted within activated domains recorded signals with significantly higher beta correlations during the visual attention trials compared with the auditory trials (Fig 5C). Our current data obtained during the stimulus-driven task are in accordance with the hypothesis [20] that strongly synchronized domains of beta activity are formed in the activated visual cortex. Similar observations of internally synchronized activation domains (local depolarizations) were reported in anaesthetized preparations [42,43,59] and more recently, in behaviorally meaningful situations [62,63,64].

### Anticipatory attention

In contrast to bottom-up processing, the beta activity that accompanied anticipatory visual attention was evenly distributed over the primary cortical areas (Fig 5D), in accordance with the results of other studies [65,66]. We assume that this activity extends down the visual hierarchy and sensitizes downstream areas via a similar beta frequency facilitation mechanism. This model is also supported by our earlier results showing that the medial suprasylvian sulcus area and downstream primary visual cortices increase their mutual synchronicity in the beta range during visually attentive situations [4]. Additionally, our results support the suggestion of Chalk and colleagues [67] that top-down attention reduces surround inhibition in the primary cortices. Such a reduction could explain the increased amplitudes of chiasm-evoked EPs (Fig 3B, right panel and Fig 4B).

### Bottom-up vs. top-down attentional mechanisms

In this experiment, we have documented that the spatial organization of the beta activity that occurs during stimulus-driven and anticipatory attentional processes clearly differs (Figs 4 and 5). Our stimulus-driven task induced a specific spatial pattern of beta (16–25 Hz) activation similar to the stimulus-specific activation map imaged with voltage-sensitive dyes in monkey primary cortex [48]. However, beta activity in spontaneously active (or weakly stimulated) primary areas had a homogeneous beta correlation distribution (0.4 ± 0.1), indicating a uniform spatial map of activity [68], similar to the homogeneous cortical activation obtained in our experiments during the anticipatory visual and auditory tasks. Such a distribution may accompany the endogenous attention that has been postulated to cause beta oscillations in a top-down cascade of visual areas ending in V1 [69].

Our current data (Fig 2) and previously published results [4,6] indicate that both attentional processes share the same mechanism of beta frequency facilitation at the synapses of recurrent pathways. Here, we hypothesize that the difference in the spatial structure of attentional beta activation is due to the lateral inhibitory loops that are activated during the visually driven paradigm. The two mechanisms together would produce similar results as “feedforward grouping circuits” proposed recently for binding ensembles of feature neurons in the visual cortex [70]. Our hypothesis is also in accordance with the model of attention modulation within the receptive fields of V4 neurons proposed by Buia and Tiesinga [71]. They found that activity in the beta frequency was synchronized during the activation of excitatory cortical cells by a strong stimulus (which caused spatial competition) and that the activity depended on feed-forward lateral inhibition. They proposed that stimulus-driven (spatial) and anticipatory (feature-based) attention is mediated by feed-forward and top-down cortical inhibitory interneurons, respectively. The physiological data obtained in our experiment suggest that their model could also apply to the primary cortex.

Several hypotheses have postulated that top-down and bottom-up attentional mechanisms differ (reviewed in [9,10]). However, most of the supporting experiments examined only the fronto-parietal pathways and overlooked activation patterns in the primary visual cortex. Here, we provide strong support for a thesis that these systems also include the primary visual cortices and that both systems utilize beta frequency activity to adjust the gain of attentional mechanisms.

## Supporting Information

S1 Fig Variability between the single-trial FFT spectra in the anticipatory paradigm. The graphs represent sets of single-trial FFT spectra obtained for all correct visual (A, n = 15) and auditory (B, n = 14) trials from an exemplary experimental day as well as for the incorrect visual (C, n = 16) trials pooled from all experimental days. The FFTs were obtained in cat E, from electrodes in area 17 (Cx 17/2). The mean FFTs from this cat are shown in Fig 2B in the main text. These examples illustrate the single-trial variability in the amount of beta activity. For better visualization, some spectra with elevated (B, C) or decreased (A) amplitudes in the beta frequency range are emphasized in red. The graphs highlight the fact that in the anticipatory paradigm, the cortical signals recorded during a small number (10–15%) of correct auditory or incorrect visual trials contained a high amount of beta activity (B, C). Conversely, in a small proportion of visual trials (A), the beta activity was not enhanced.
(PDF)

S2 Fig Relative visual beta amplitudes. For each recording site, the task (stimulus-driven vs. anticipatory) and trial modality (visual vs. auditory) beta amplitude was calculated by averaging the spectral values within the 16–24 Hz frequency range (in FFTs averaged from three experimental days). The relative visual beta amplitudes (ordinate) were then measured by subtracting the auditory beta amplitude from the visual amplitude, dividing the result by the respective auditory beta amplitude and multiplying it by 100% (see the Methods section). Each box plot represents the consolidated data from all electrodes in all cats trained in a given paradigm. The results of the graph show that the beta signals in the visual trials were significantly stronger than those in the auditory trials, both for the stimulus-induced (by 20.3 ± 3.9% on average, n = 8) and anticipatory (by 6 ± 1%, n = 13) attention tasks (P < 0.001 for both comparisons, t-tests). Larger variability for the stimulus-driven paradigm results from the large differences between individual recording sites in the strength of the beta signals that increased during visual trials (spatial patchiness).
(PDF)

S3 Fig Relative amplitude differences of the evoked potentials in different beta states during the tasks of visual and auditory modalities. EPs induced by chiasmatic stimulation during visual and auditory trials were analyzed in all recording sites and all cats in the two experimental paradigms (cf. Table 1). Each box shows the normalized percentage difference (Δ%) between the EP amplitudes calculated for the appropriate recording site with the formula Δ% = 100% x (vis high − aud low) / aud low. In this formula “vis high” indicates the mean amplitude of the EPs that were preceded by bursts of high beta activity in the visual trials and”aud low” refers to the mean amplitude of the EPs that were preceded by low beta activity in the auditory trials. Black boxes = vis high < aud low, gray boxes = vis high > aud low. For each paradigm mean percentage difference (with SEM) was obtained from all available sites. P indicates the significance of the average amplitude change in the relevant table (one-sample t-test).
(PDF)

S4 Fig Synchronization of beta activity in different areas of the visual cortex. A, B. Box plots of the maximal cross-correlation values (referred to as correlation) between the beta signals recorded at different sites in the visual cortex during the stimulus-driven (A) and anticipatory (B) attentional paradigms. Red boxes—both recording sites in area 17; blue—both sites in area 18; green—one site/electrode in area 17, the other in area 18; black—all site pairs grouped together (n = number of site pairs). The left-hand box in each color indicates the correlations obtained during the visual task (V), and the right-hand box indicates those obtained during the auditory task (A). The bottom and the top of the box represent the first and the third quartiles of the data range, respectively. The whiskers indicate the minimum and maximum of the data. The median and the mean are indicated by the horizontal line and the plus sign, respectively, inside the box. Significant differences between individual mean values (as shown by brackets above) are denoted by asterisks (* for P ≤ 0.05, ** for P ≤ 0.01 and *** for P ≤ 0.001; t-tests with Holm-Bonferroni correction). C, D. The differences between the visual and auditory correlations obtained for individual pairs of sites are plotted against the respective visual correlation values in the stimulus-driven (C) and anticipatory (D) situations. The filled symbols indicate significant correlation differences for individual electrode pairs (P ≤ 0.05, t-test). In case of four data points, the time lag values (τ, in ms) are indicated in brackets for the visual (first value) and auditory (second value) cross-correlograms (see the text for details).
(PDF)

S5 Fig Synchronization of beta signals for individual pairs of electrodes. Each data point (square) represents the visual (ordinate) and auditory (abscissa) correlation between the beta signals recorded from a single pair of electrodes. For the entire group of electrode pairs, the visual and auditory correlation values related well with each other during the stimulus-driven task (red squares; Pearson r = 0.972, P < 0.001, n = 20, 95% confidence interval 0.928 to 0.989), and this relationship was almost perfectly linear during the anticipatory task (blue squares; Pearson r = 0.996, P < 0.001, n = 61, 95% confidence interval 0.993 to 0.998), with the regression line Y = 0.994*X + 0.001 (with respective 95% confidence intervals: 0.971 to 1.017 and -0.014 to 0.015) that was not different from the diagonal. However, 75% of the data points for the stimulus-driven task are located below the diagonal, leading to a steeper regression line Y = 1.223*X − 0.233 (respective 95% confidence intervals: 1.075 to 1.371 and -0.3421 to -0.1241), with a positive X intercept of 0.191 (95% confidence intervals: 0.115 to 0.251).
(PDF)

S6 Fig Correlation during incorrect visual trials in stimulus-driven paradigm. The graph shows differences between correlations from incorrect visual trials and correlations from correct auditory trials (ordinate) plotted against the respective correlation values from incorrect visual trials (abscissa). No relation between both variables was detected (r = -0.21, P = 0.38, n = 20). Red circles—both recording sites in area 17; blue—both sites in area 18; green—one site in area 17, other in area 18. Due to the fact that the same moving visual stimulus was present during correct and incorrect visual trials, this analysis confirms that the correlation pattern observed for correct trials (S4C Fig) was caused mainly by the attentional processes and not by the presence of the stimulus.
(PDF)

S1 Text Detailed analysis of S4 Fig. This text presents the detailed analysis of synchronization of beta activity in different areas of the visual cortex shown in S4 Fig.
(PDF)

## References

[1] Wang X-J. Neurophysiological and computational principles of cortical rhythms in cognition. Physiol Rev. 2010;90: 1195–1268. 10.1152/physrev.00035.2008 20664082 PMC2923921

[2] Wróbel A. Attentional Activation in Corticothalamic Loops of the Visual System In: Werner JS, Chalupa LM, editors. The New Visual Neurosciences. Cambridge (MA): MIT Press; 2014 pp. 339–349.

[3] Kastner S, Pinsk MA, De Weerd P, Desimone R, Ungerleider LG. Increased activity in human visual cortex during directed attention in the absence of visual stimulation. Neuron. 1999;22: 751–761. 10230795 10.1016/s0896-6273(00)80734-5

[4] Wróbel A, Ghazaryan A, Bekisz M, Bogdan W, Kamiński J. Two streams of attention dependent beta activity in the striate recipient zone of cat's lateral posterior—pulvinar complex. J Neurosci. 2007;27: 2230–2240. 17329420 10.1523/JNEUROSCI.4004-06.2007 PMC6673477

[5] Saalmann YB, Pinsk MA, Wang L, Li X, Kastner S. The pulvinar regulates information transmission between cortical areas based on attention demands. Science. 2013;337: 753–756. 10.1126/science.1223082 PMC3714098 22879517

[6] Bekisz M, Wróbel A. 20 Hz rhythm of activity in visual system of perceiving cat. Acta Neurobiol Exp (Warsz). 1993;53: 175–182. 8317245

[7] Zhang X, Zhaoping L, Zhou T, Fang F. Neural activities in V1 create a bottom-up saliency map. Neuron. 2012;73: 183–192. 10.1016/j.neuron.2011.10.035 22243756

[8] Saalmann YB, Kastner S. Cognitive and perceptual functions of the visual thalamus. Neuron. 2011;71: 209–223. 10.1016/j.neuron.2011.06.027 21791281 PMC3148184

[9] Corbetta M, Shulman GL. Control of goal-directed and stimulus-driven attention in the brain. Nat Rev Neurosci. 2002;3: 201–215. 11994752 10.1038/nrn755

[10] Chica AB, Bartolomeo P, Lupiánez J. Two cognitive and neural systems for endogenous and exogenous spatial attention. Behav Brain Res. 2013;237: 107–123. 10.1016/j.bbr.2012.09.027 23000534

[11] Bastos AM, Vezoli J, Bosman CA, Schoffelen J-M, Oostenveld R, Dowdall JR, et al Visual areas exert feedforward and feedback influences through distinct frequency channels. Neuron. 2015;85: 390–401. 10.1016/j.neuron.2014.12.018 25556836

[12] Wróbel A, Bekisz M, Kublik E, Waleszczyk W. 20 Hz bursting beta activity in the cortico-thalamic system of visually attending cats. Acta Neurobiol Exp (Warsz). 1994;54: 95–107. 8053417

[13] Lindström S, Wróbel A. Frequency dependent corticofugal excitation of principal cells in the cat's dorsal lateral geniculate nucleus. Exp Brain Res. 1990a;79: 313–318. 2323378 10.1007/BF00608240

[14] von Stein A, Chiang C, König P. Top-down processing mediated by interareal synchronization. Proc Natl Acad Sci USA. 2000;97: 14748–14753. 11121074 10.1073/pnas.97.26.14748 PMC18990

[15] Gross J, Schmitz F, Schnitzler I, Kessler K, Shapiro K, Hommel B, et al Modulation of long-range neural synchrony reflects temporal limitations of visual attention in humans. Proc Natl Acad Sci USA. 2004;101: 13050–13055. 15328408 10.1073/pnas.0404944101 PMC516515

[16] Buschman TJ, Miller EK. Top-down versus bottom-up control of attention in the prefrontal and posterior parietal cortices. Science. 2007;315: 1860–1862. 17395832 10.1126/science.1138071

[17] Saalmann YB, Pigarev IN, Vidyasagar TR. Neural mechanisms of visual attention: how top-down feedback highlights relevant locations. Science. 2007;316: 1612–1615. 17569863 10.1126/science.1139140

[18] Pesaran B, Nelson MJ, Andersen RA. Free choice activates a decision circuit between frontal and parietal cortex. Nature. 2008;453: 406–409. 10.1038/nature06849 18418380 PMC2728060

[19] Haenschel C, Baldeweg T, Croft RJ, Whittington M, Gruzelier J. Gamma and beta frequency oscillations in response to novel auditory stimuli: a comparison of human electroencephalogram (EEG) data with *in vitro* models. Proc Natl Acad Sci USA. 2000;97: 7645–7650. 10852953 10.1073/pnas.120162397 PMC16599

[20] Wróbel A. Beta activity: a carrier for visual attention. Acta Neurobiol Exp (Warsz). 2000;60: 247–260. 10909182 10.55782/ane-2000-1344

[21] Tusa RJ, Palmer LA, Rosenquist AC. The retinotopic organization of area 17 (striate cortex) in the cat. J Comp Neurol. 1978;177: 213–235. 413845 10.1002/cne.901770204

[22] Tusa RJ, Rosenquist AC, Palmer LA. Retinotopic organization of areas 18 and 19 in the cat. J Comp Neurol. 1979;185: 657–678. 447876 10.1002/cne.901850405

[23] Garey LJ. A Light and Electron Microscopic Study of the Visual Cortex of the Cat and Monkey. Proc R Soc Lond B Biol Sci. 1971;179: 21–40. 4398773 10.1098/rspb.1971.0079

[24] Innocenti GM, Manger PR, Masiello I, Colin I, Tettoni L. Architecture and callosal connections of visual areas 17, 18, 19 and 21 in the ferret (Mustela putorius). Cereb Cortex. 2002;12: 411–422. 11884356 10.1093/cercor/12.4.411

[25] Roelfsema PR, Engel AK, Konig P, Singer W. Visuomotor integration is associated with zero time-lag synchronization among cortical areas. Nature. 1997;385: 157–161. 8990118 10.1038/385157a0

[26] Bekisz M, Wróbel A. Attention-dependent coupling between beta activities recorded in the cat's thalamic and cortical representations of the central visual field. Eur J Neurosci. 2003;17: 421–426. 12542680 10.1046/j.1460-9568.2003.02454.x

[27] Mitzdorf U, Singer W. Monocular activation of visual cortex in normal and monocularly deprived cats: an analysis of evoked potentials J Physiol (Lond). 1980;304: 203–220. 7441534 10.1113/jphysiol.1980.sp013320 PMC1282926

[28] Ferster D, Lindström S. An intracellular analysis of geniculo-cortical connectivity in area 17 of the cat. J Physiol (Lond). 1983;342: 181–215. 6631731 10.1113/jphysiol.1983.sp014846 PMC1193954

[29] Lindström S, Wróbel A. Private inhibitory systems for the X and Y pathways in the dorsal lateral geniculate nucleus of the cat. J Physiol (Lond). 1990b;429: 259–280. 2277347 10.1113/jphysiol.1990.sp018255 PMC1181698

[30] Okun M, Lampl I. Instantaneous correlation of excitation and inhibition during ongoing and sensory-evoked activities. Nat Neurosci. 2008;11: 535–537. 10.1038/nn.2105 18376400

[31] Arieli A, Sterkin A, Grinvald A, Aertsen A. Dynamics of ongoing activity: explanation of the large variability in evoked cortical responses. Science. 1996;273: 1868–1871. 8791593 10.1126/science.273.5283.1868

[32] Wróbel A, Kublik E. Modification of evoked potentials in the rat's barrel cortex induced by conditioning stimuli In: Kossut M, editor. Plasticity of the barrel cortex. New York: Graham Publ Corp; 2000 pp. 229–239.

[33] Benjamini Y, Hochberg Y. Controlling the false discovery rate: a practical and powerful approach to multiple testing. J R Statist Soc B. 1995;57: 289–300.

[34] Bekisz M, Wróbel A. Coupling of beta and gamma activity in cortico-thalamic system of cats attending to visual stimuli. Neuroreport. 1999;10: 3589–3594. 10619649 10.1097/00001756-199911260-00023

[35] Kublik E, Musial P, Wróbel A. Identification of principal components in cortical evoked potentials by brief surface cooling. Clin Neurophysiol. 2001;112: 1720–1725. 11514255 10.1016/s1388-2457(01)00603-4

[36] Buffalo EA, Fries P, Desimone R. Layer-specific attentional modulation in early visual areas. 2004; Program No. 717.6 Abstract Viewer/Itinerary Planner. Washington, DC: Society for Neuroscience 2004.

[37] Buffalo EA, Fries P, Landman R, Buschman TJ, Desimone R. Laminar differences in gamma and alpha coherence in the ventral stream. Proc Natl Acad Sci USA. 2011;108: 11262–11267. 10.1073/pnas.1011284108 21690410 PMC3131344

[38] van Aerde KI, Mann EO, Canto CB, Heistek TS, Linkenkaer-Hansen K, Mulder AB, et al Flexible spike timing of layer 5 neurons during dynamic beta oscillation shifts in rat prefrontal cortex. J Physiol (Lond). 2009;587: 5177–5196. 19752121 10.1113/jphysiol.2009.178384 PMC2790257

[39] Maier A, Adams GK, Aura Ch, Leopold DA. Distinct superficial and deep laminar domains of activity in the visual cortex during rest and stimulation. Front Syst Neurosci. 2010;4: 1–11. 20802856 10.3389/fnsys.2010.00031 PMC2928665

[40] Maier A, Aura Ch, Leopold DA. Infragranular sources of sustained local field potential responses in macaque primary visual cortex. J Neurosci. 2011;31: 1971–1980. 10.1523/JNEUROSCI.5300-09.2011 21307235 PMC3075009

[41] den Ouden HEM, Kok P, de Lange FP. How prediction errors shape perception, attention, and motivation. Front Psychol. 2012;3: 548 10.3389/fpsyg.2012.00548 23248610 PMC3518876

[42] Roland PE. Dynamic depolarization fields in the cerebral cortex. Trends Neurosci. 2002;25: 183–191. 11998686 10.1016/s0166-2236(00)02125-1

[43] Roland PE. Six principles of visual cortical dynamics. Front Syst Neurosci. 2010;4: 28 10.3389/fnsys.2010.00028 20661451 PMC2906257

[44] Chen Y, Geisler WS, Seidemann E. Optimal decoding of correlated neural population responses in the primate visual cortex. Nat Neurosci. 2006;9: 1412–1420. 17057706 10.1038/nn1792 PMC1851689

[45] Nauhaus I, Busse L, Carandini M, Ringach DL. Stimulus contrast modulates functional connectivity in visual cortex. Nat Neurosci. 2009;12: 70–76. 10.1038/nn.2232 19029885 PMC2610236

[46] Ozeki H, Finn IM, Schaffer ES, Miller KD, Ferster D. Inhibitory stabilization of the cortical network underlies visual surround suppression. Neuron. 2009;62: 578–592. 10.1016/j.neuron.2009.03.028 19477158 PMC2691725

[47] Sakata S, Harris KD. Laminar structure of spontaneous and sensory-evoked population activity in auditory cortex. Neuron. 2009;64: 404–418. 10.1016/j.neuron.2009.09.020 19914188 PMC2778614

[48] Ayzenshtat I, Meirovithz E, Edelman H, Werner-Reiss U, Bienenstock E, Abeles M, et al Precise spatiotemporal patterns among visual cortical areas and their relation to visual stimulus processing. J Neurosci. 2010;30: 11232–11245. 10.1523/JNEUROSCI.5177-09.2010 20720131 PMC6633472

[49] Jiang X, Wang G, Lee AJ, Stornetta RL, Zhu JJ. The organization of two new cortical interneuronal circuits. Nat Neurosci. 2013;16: 210–218. 10.1038/nn.3305 23313910 PMC3589105

[50] Ramalingam N, McManus JNJ, Li W, Gilbert CD. Top-down modulation of lateral interactions in visual cortex. J Neurosci. 2013;33: 1773–1789. 10.1523/JNEUROSCI.3825-12.2013 23365217 PMC3711382

[51] Bharmauria V, Bachatene L, Cattan AS, Rouat AJ, Molotchnikoff S. Synergistic activity between primary visual neurons. Neuroscience. 2014;268: 255–264. 10.1016/j.neuroscience.2014.03.027 24662850

[52] Harris KD, Mrsic-Flogel TD. Cortical connectivity and sensory coding. Nature. 2013;503: 51–56. 10.1038/nature12654 24201278

[53] Castro-Alamancos MA. Role of thalamocortical sensory suppression during arousal: focusing sensory inputs in neocortex. J Neurosci. 2002;22: 9651–9655. 12427819 10.1523/JNEUROSCI.22-22-09651.2002 PMC6757812

[54] Cutzu F, Tsotsos JK. The selective tuning model of attention: psychophysical evidence for a suppressive annulus around an attended item. Vision Research. 2003;43: 205–219. 12536142 10.1016/s0042-6989(02)00491-1

[55] Ferezou I, Haiss F, Gentet LJ, Aronoff R, Weber B, Petersen CCH. Spatiotemporal dynamics of cortical sensorimotor integration in behaving mice. Neuron. 2007;56: 907–923. 18054865 10.1016/j.neuron.2007.10.007

[56] Poulet JF, Petersen CC. Internal brain state regulates membrane potential synchrony in barrel cortex of behaving mice. Nature. 2008;454: 881–885. 10.1038/nature07150 18633351

[57] Lee Ch-Ch, Middlebrooks JC. Auditory cortex spatial sensitivity sharpens during task performance. Nat Neurosci. 2011;14: 108–114. 10.1038/nn.2713 21151120 PMC3076022

[58] Schmiedt JT, Maier A, Fries P, Saunders RC, Leopold DA, Schmid MC. Beta oscillation dynamics in extrastriate cortex after removal of primary visual cortex. J Neurosci. 2014;34: 11864–11857. 10.1523/JNEUROSCI.0509-14.2014 PMC4145181 25164679

[59] Ahissar M, Ahissar E, Bergman H, Vaadia E. Encoding of sound-source location and movement activity of single neurons and interactions between adjacent neurons in the monkey auditory cortex. J Neurophysiol. 1992;67: 203–215. 1552320 10.1152/jn.1992.67.1.203

[60] Arieli A, Shoham D, Hildesheim R, Grinvald A. Coherent spatiotemporal patterns of ongoing activity revealed by real-time optical imaging coupled with single-unit recording in the cat visual cortex. J Neurophysiol. 1995;73: 2072–2093. 7623099 10.1152/jn.1995.73.5.2072

[61] Maris E, Womelsdorf T, Desimone R, Fries P. Rhythmic neuronal synchronization in visual cortex entails spatial phase relation diversity that is modulated by stimulation and attention. NeuroImage. 2013;74: 99–116. 10.1016/j.neuroimage.2013.02.007 23416733 PMC3618893

[62] Lashgari R, Li X, Chen Y, Kremkow J, Bereshpolova Y, Swadlow HA, et al Response properties of local field potentials and neighboring single neurons in awake primary visual cortex. J Neurosci. 2012;32: 11396–11413. 10.1523/JNEUROSCI.0429-12.2012 22895722 PMC3436073

[63] Chu CCJ, Chien PF, Hung CP. Tuning dissimilarity explains short distance decline of spontaneous spike correlation in macaque V1. Vis Res. 2014;96: 113–132. 10.1016/j.visres.2014.01.008 24486852

[64] Ruff DA, Cohen MR. Attention can either increase or decrease spike count. Nature Neurosci. 2014;17: 1591–1598. 10.1038/nn.3835 25306550 PMC4446056

[65] Siegel M, König P. A functional gamma-band defined by stimulus-dependent synchronization in area 18 of awake behaving cats. J Neurosci. 2003;23: 4251–4260. 12764113 10.1523/JNEUROSCI.23-10-04251.2003 PMC6741080

[66] Schroeder CE, Lakatos P. Low-frequency neuronal oscillations as instruments of sensory selection. Trends Neurosci. 2009;32: 9–18. 10.1016/j.tins.2008.09.012 19012975 PMC2990947

[67] Chalk M, Herrero JL, Gieselmann MA, Delicato LS, Gotthardt S, Thiele A. Attention reduces stimulus-driven gamma frequency oscillations and spike field coherence in V1. Neuron. 2010;66: 114–125. 10.1016/j.neuron.2010.03.013 20399733 PMC2923752

[68] Leopold DA, Logothetis NK. Spatial patterns of spontaneous local field activity in the monkey visual cortex. Nat Rev Neurosci. 2003;14: 195–205. 10.1515/revneuro.2003.14.1-2.195 12929926

[69] Buffalo EA, Fries P, Landman R, Liang H, Desimone R. A backward progression of attentional effects in the ventral stream. Proc Natl Acad Sci USA. 2010;107: 361–365. 10.1073/pnas.0907658106 20007766 PMC2806732

[70] Martin AB, von der Heydt R. Spike synchrony reveals emergence of proto-objects in visual cortex. J Neurosci. 2015;35: 6860–6870. 10.1523/JNEUROSCI.3590-14.2015 25926461 PMC4412900

[71] Buia CI, Tiesinga PH. Role of interneuron diversity in the cortical microcircuit for attention. J Neurophysiol. 2008;99: 2158–2182. 10.1152/jn.01004.2007 18287553

