# Supplementary figures and images for: The Primary Visual Cortex Is Differentially Modulated by Stimulus-Driven and Top-Down Attention
Source: PLoS One. 2016 Jan 5;11(1):e0145379. doi: 10.1371/journal.pone.0145379 (PMC4701232; doi:10.1371/journal.pone.0145379)

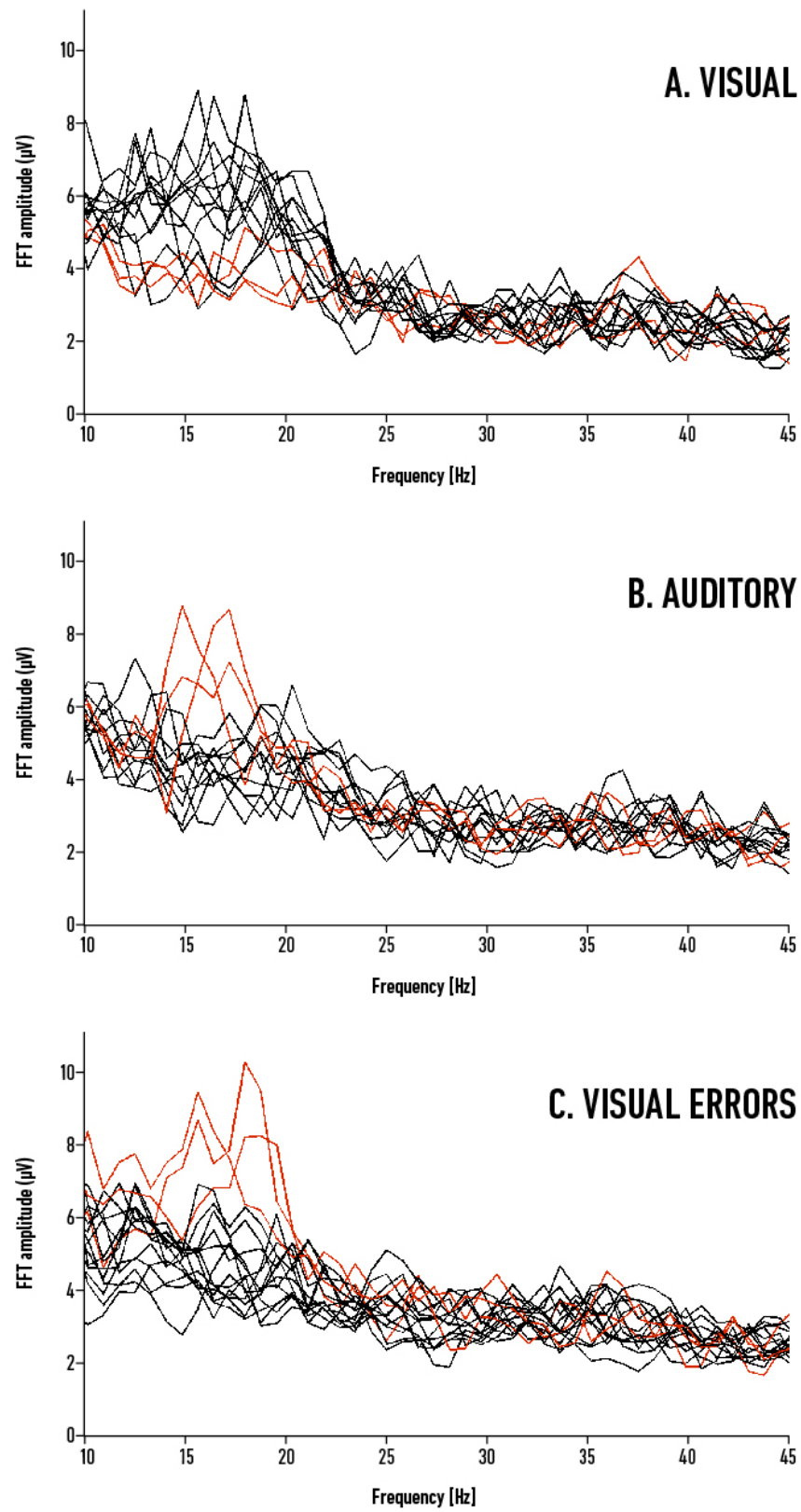

**S1 Fig. Variability between the single-trial FFT spectra in the anticipatory paradigm.**

Supplement: S1 Fig — The graphs represent sets of single-trial FFT spectra obtained for all correct visual (A, n = 15) and auditory (B, n = 14) trials from an exemplary experimental day as well as for the incorrect visual (C, n = 16) trials pooled from all experimental days. The FFTs were obtained in cat E, from electrodes in area 17 (Cx 17/2). The mean FFTs from this cat are shown in Fig 2B in the main text. These examples illustrate the single-trial variability in the amount of beta activity. For better visualization, some spectra with elevated (B, C) or decreased (A) amplitudes in the beta frequency range are emphasized in red. The graphs highlight the fact that in the anticipatory paradigm, the cortical signals recorded during a small number (10–15%) of correct auditory or incorrect visual trials contained a high amount of beta activity (B, C). Conversely, in a small proportion of visual trials (A), the beta activity was not enhanced. (PDF) [file pone.0145379.s001.pdf]

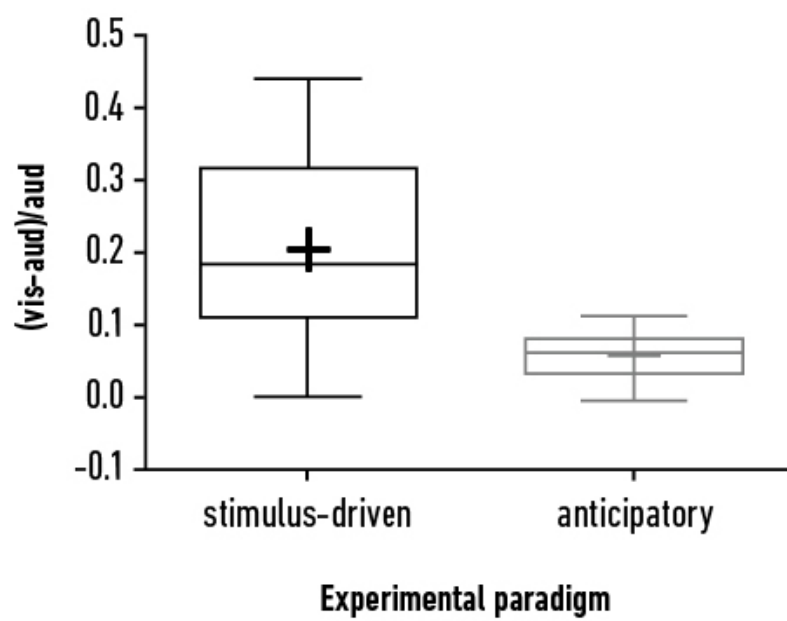

**S2 Fig. Relative visual beta amplitudes.**

Supplement: S2 Fig — For each recording site, the task (stimulus-driven vs. anticipatory) and trial modality (visual vs. auditory) beta amplitude was calculated by averaging the spectral values within the 16–24 Hz frequency range (in FFTs averaged from three experimental days). The relative visual beta amplitudes (ordinate) were then measured by subtracting the auditory beta amplitude from the visual amplitude, dividing the result by the respective auditory beta amplitude and multiplying it by 100% (see the Methods section). Each box plot represents the consolidated data from all electrodes in all cats trained in a given paradigm. The results of the graph show that the beta signals in the visual trials were significantly stronger than those in the auditory trials, both for the stimulus-induced (by 20.3 ± 3.9% on average, n = 8) and anticipatory (by 6 ± 1%, n = 13) attention tasks (P < 0.001 for both comparisons, t-tests). Larger variability for the stimulus-driven paradigm results from the large differences between individual recording sites in the strength of the beta signals that increased during visual trials (spatial patchiness). (PDF) [file pone.0145379.s002.pdf]

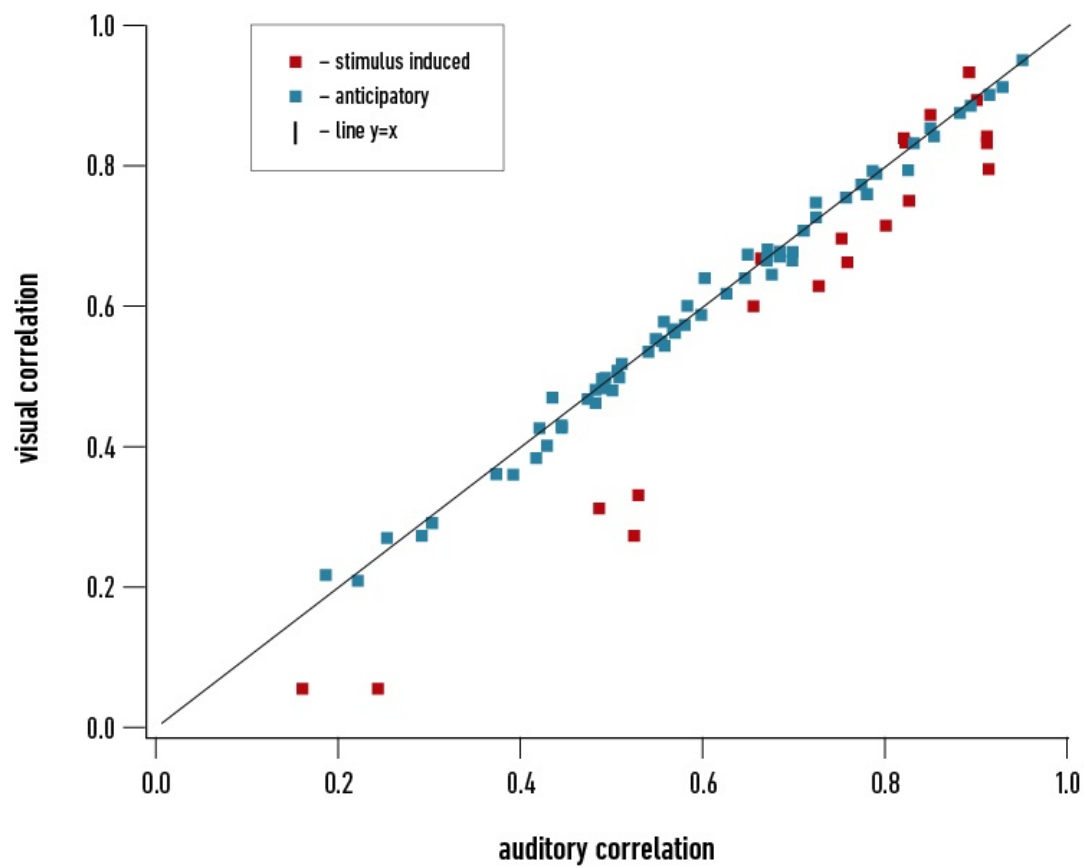

**S5 Fig. Synchronization of beta signals for individual pairs of electrodes.**

Supplement: S5 Fig — Each data point (square) represents the visual (ordinate) and auditory (abscissa) correlation between the beta signals recorded from a single pair of electrodes. For the entire group of electrode pairs, the visual and auditory correlation values related well with each other during the stimulus-driven task (red squares; Pearson r = 0.972, P < 0.001, n = 20, 95% confidence interval 0.928 to 0.989), and this relationship was almost perfectly linear during the anticipatory task (blue squares; Pearson r = 0.996, P < 0.001, n = 61, 95% confidence interval 0.993 to 0.998), with the regression line Y = 0.994*X + 0.001 (with respective 95% confidence intervals: 0.971 to 1.017 and -0.014 to 0.015) that was not different from the diagonal. However, 75% of the data points for the stimulus-driven task are located below the diagonal, leading to a steeper regression line Y = 1.223*X − 0.233 (respective 95% confidence intervals: 1.075 to 1.371 and -0.3421 to -0.1241), with a positive X intercept of 0.191 (95% confidence intervals: 0.115 to 0.251). (PDF) [file pone.0145379.s005.pdf]

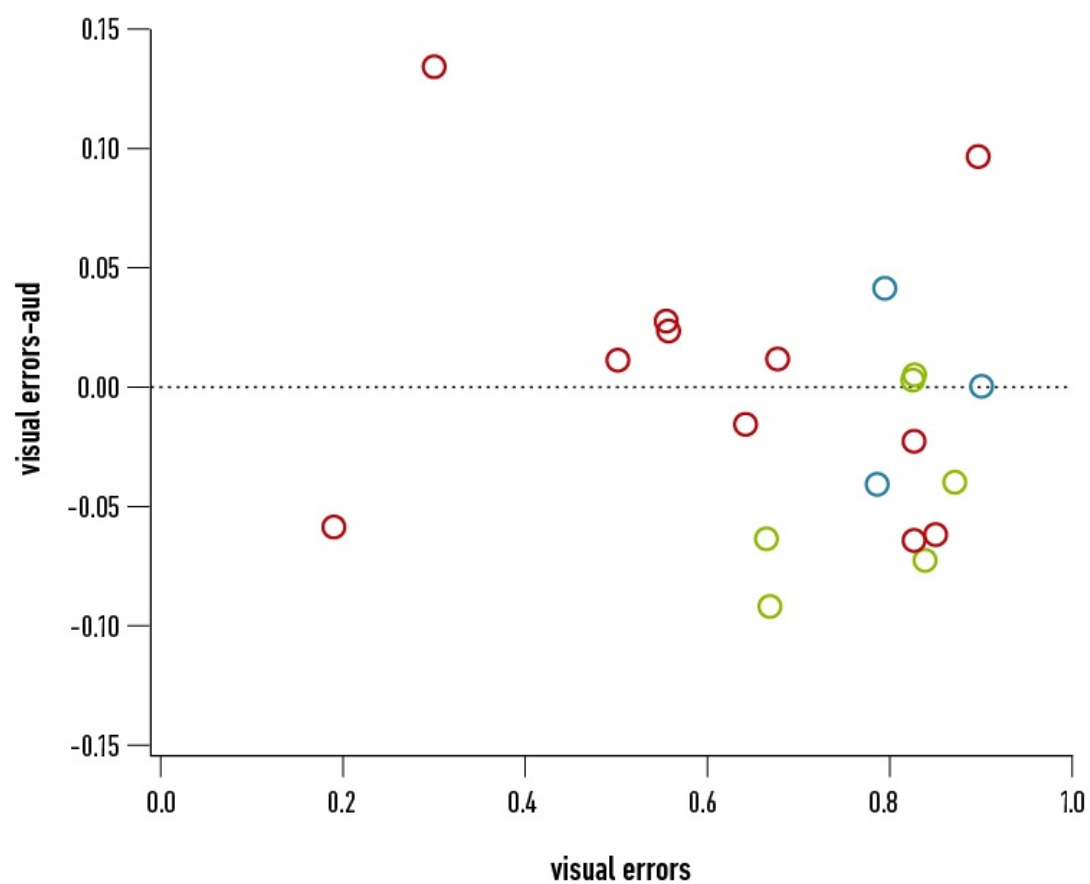

**S6 Fig. Correlation during incorrect visual trials in stimulus-driven paradigm.**

Supplement: S6 Fig — The graph shows differences between correlations from incorrect visual trials and correlations from correct auditory trials (ordinate) plotted against the respective correlation values from incorrect visual trials (abscissa). No relation between both variables was detected (r = -0.21, P = 0.38, n = 20). Red circles—both recording sites in area 17; blue—both sites in area 18; green—one site in area 17, other in area 18. Due to the fact that the same moving visual stimulus was present during correct and incorrect visual trials, this analysis confirms that the correlation pattern observed for correct trials (S4C Fig) was caused mainly by the attentional processes and not by the presence of the stimulus. (PDF) [file pone.0145379.s006.pdf]
