# Supplementary material for: The Primary Visual Cortex Is Differentially Modulated by Stimulus-Driven and Top-Down Attention
Source: PLoS One. 2016 Jan 5;11(1):e0145379. doi: 10.1371/journal.pone.0145379 (PMC4701232; doi:10.1371/journal.pone.0145379)
Supplement: S3 Fig — EPs induced by chiasmatic stimulation during visual and auditory trials were analyzed in all recording sites and all cats in the two experimental paradigms (cf. Table 1). Each box shows the normalized percentage difference (Δ%) between the EP amplitudes calculated for the appropriate recording site with the formula Δ% = 100% x (vis high − aud low) / aud low. In this formula “vis high” indicates the mean amplitude of the EPs that were preceded by bursts of high beta activity in the visual trials and”aud low” refers to the mean amplitude of the EPs that were preceded by low beta activity in the auditory trials. Black boxes = vis high < aud low, gray boxes = vis high > aud low. For each paradigm mean percentage difference (with SEM) was obtained from all available sites. P indicates the significance of the average amplitude change in the relevant table (one-sample t-test). (PDF) [file pone.0145379.s003.pdf]

(vis high – aud low) / aud low

A. STIMULUS-DRIVEN ATTENTION

	Cx18/1	Cx17/1	Cx18/2
Cat A	-12.69	1.22	-15.00
	Cx17/1	Cx17/2	Cx17/3
Cat B	-14.61	-20.41	0.63
	Cx18/1	Cx18/2	
Cat F-p1	-2.18	-14.14	

Mean=-9.6 ± 2.9 P=0.01 (from zero)

B. ANTICIPATORY ATTENTION

	Cx17/1	Cx17/2	Cx17/3	
Cat C	18.14	17.79	29.82	
	Cx17/1	Cx18/1	Cx17/2	Cx18/2
Cat D	5.78	6.15	6.04	14.36
	Cx17/1	Cx17/2	Cx18/1	Cx18/2
Cat E	7.54	1.17	1.92	4.23
	Cx18/1	Cx18/2		
Cat F-p2	11.27	2.70		

Mean=9.8 ± 2.3 P=0.001 (from zero)

S3 Fig. Relative amplitude differences of the evoked potentials in different beta states during the tasks of visual and auditory modalities
