# Supplementary material for: The Primary Visual Cortex Is Differentially Modulated by Stimulus-Driven and Top-Down Attention
Source: PLoS One. 2016 Jan 5;11(1):e0145379. doi: 10.1371/journal.pone.0145379 (PMC4701232; doi:10.1371/journal.pone.0145379)
Supplement: S4 Fig — A, B. Box plots of the maximal cross-correlation values (referred to as correlation) between the beta signals recorded at different sites in the visual cortex during the stimulus-driven (A) and anticipatory (B) attentional paradigms. Red boxes—both recording sites in area 17; blue—both sites in area 18; green—one site/electrode in area 17, the other in area 18; black—all site pairs grouped together (n = number of site pairs). The left-hand box in each color indicates the correlations obtained during the visual task (V), and the right-hand box indicates those obtained during the auditory task (A). The bottom and the top of the box represent the first and the third quartiles of the data range, respectively. The whiskers indicate the minimum and maximum of the data. The median and the mean are indicated by the horizontal line and the plus sign, respectively, inside the box. Significant differences between individual mean values (as shown by brackets above) are denoted by asterisks (* for P ≤ 0.05, ** for P ≤ 0.01 and *** for P ≤ 0.001; t-tests with Holm-Bonferroni correction). C, D. The differences between the visual and auditory correlations obtained for individual pairs of sites are plotted against the respective visual correlation values in the stimulus-driven (C) and anticipatory (D) situations. The filled symbols indicate significant correlation differences for individual electrode pairs (P ≤ 0.05, t-test). In case of four data points, the time lag values (τ, in ms) are indicated in brackets for the visual (first value) and auditory (second value) cross-correlograms (see the text for details). (PDF) [file pone.0145379.s004.pdf]

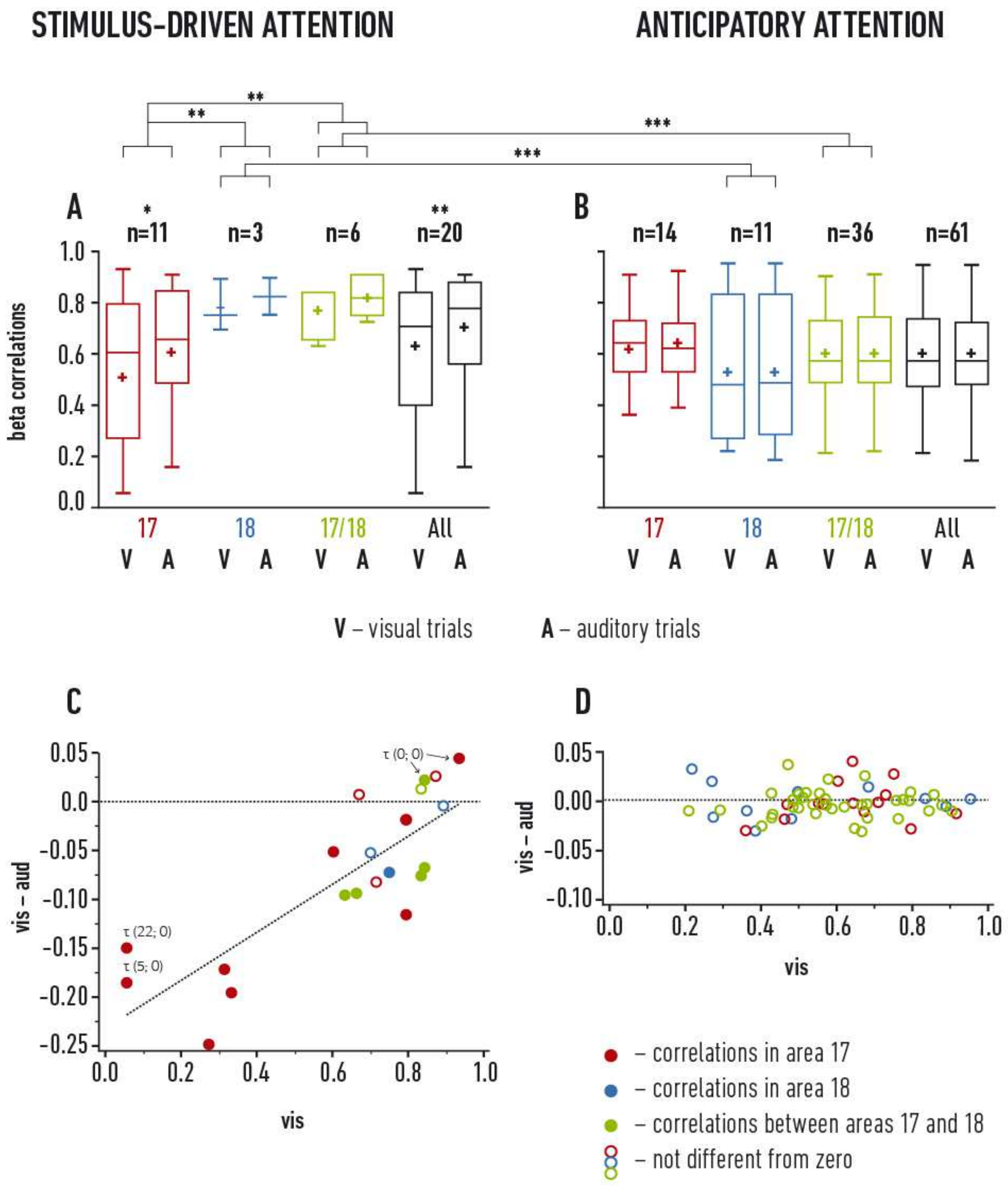

S4 Fig. Synchronization of beta activity in different areas of the visual cortex.
