# Supplementary material for: The Primary Visual Cortex Is Differentially Modulated by Stimulus-Driven and Top-Down Attention
Source: PLoS One. 2016 Jan 5;11(1):e0145379. doi: 10.1371/journal.pone.0145379 (PMC4701232; doi:10.1371/journal.pone.0145379)
Supplement: S1 Text — This text presents the detailed analysis of synchronization of beta activity in different areas of the visual cortex shown in S4 Fig. (PDF) [file pone.0145379.s007.pdf]

S1 Text. Detailed analysis of S4 Fig.

In the stimulus-driven paradigm (A), a significant difference was found between the visual and auditory correlations calculated from pairs of signals recorded exclusively within area 17 (0.51 ± 0.1 for visual and 0.61 ± 0.08 for auditory trials; n = 11 pairs, P = 0.02, paired t-test with Holm-Bonferroni correction). A similar trend was present for the recording pairs in area 18 (0.78 ± 0.06 vs. 0.83 ± 0.04) and for mixed recordings between areas 17/18 (0.77 ± 0.04 vs. 0.82 ± 0.03). Moreover, in this paradigm, all of the correlations (both modalities pooled together) calculated for pairs of signals recorded within area 17 (0.56 ± 0.06, n = 22 pairs) were, on average, smaller than those calculated from area 18 (0.8 ± 0.03, n = 6 pairs; P = 0.003, unpaired t-test) or between areas 17 and 18 (0.8 ± 0.03, n = 12 correlations; P = 0.004, unpaired t-test) All of the correlations within area 17 had also higher variances than the correlations from area 18 (F-test, P = 0.02) or from mixed 17/18 pairs (F-test, P = 0.001; all probabilities with Holm-Bonferroni corrections). This higher variance was due to the large differences between the correlations measured at different electrode pairs in area 17, predominantly during the visual task.

In contrast, during the anticipatory task (B) the measured correlations were similar during the visual and auditory attention tasks and for the visual cortical areas. The respective modality mean values (and SEMs) also did not differ. Therefore, we listed only single values for each group: 0.63 ± 0.04 for electrodes placed in area 17 (P = 0.7, n = 14), 0.53 ± 0.08 for area 18 (P = 0.9, n = 11) and 0.6 ± 0.04 for mixed pairs from areas 17/18 (P = 0.15, n = 36, paired t-tests).

It is interesting to note that during the stimulus-driven paradigm, the largest spatial correlation variance was obtained for area 17, while in the anticipatory attention paradigm, we observed significantly larger variances for all correlations within area 18 compared with those recorded from area 17 or between areas 17 and 18 (A, B; P ≤ 0.01 for both comparisons, F-tests). Additionally, all correlations within area 18 (0.53 ± 0.06, n = 22 pairs) or between areas 17 and 18 (0.6 ± 0.02, n = 72 pairs) in the anticipatory paradigm were, on average, weaker than the corresponding correlations during the stimulus-induced task (0.8 ± 0.03, n = 6 pairs; P = 0.0009 and 0.8 ± 0.03, n = 12 pairs; P = 0.0004, respectively, unpaired t-tests).

Note that the data points in parts C and D, with low (< 0.4) correlations in the stimulus-driven paradigm, were obtained solely in area 17 (as we lack data from area 18). Despite this, the differences between the visual and auditory correlations for pairs from area 18 and areas 17/18 are also significantly related to the visual correlation values (r = 0.69, P = 0.04, n = 9). This suggests that in the stimulus-driven task, the beta correlation patterns are modality dependent within both areas 17 and 18. In contrast, during anticipatory attention, the difference between the visual and auditory correlations does not depend on the visual correlation strength for any of the data groups: sites located exclusively in area 17 (r = 0.18, P = 0.53, n = 14), in area 18 (r = -0.05, P = 0.88, n = 11) or mixed pairs from two different cortical areas (r = 0.01, P = 0.93, n = 36).
